# Functional divergence within mulberry-associated *Pantoea ananatis*: Insights from comparative genomics and host-contact metabolomics

**DOI:** 10.1080/21505594.2026.2749997

**Published:** 2026-11-07

**Authors:** Ting Yuan, Peijia Yang, Xinpeng Huang, Rumei Li, Yuanling Liu, Menying Huang, Jinhao Li, Huiyu Yi, Jiping Liu

**Affiliations:** Guangdong Provincial Key Lab of Agro-Animal Genomics and Molecular Breeding, College of Animal Science, South China Agricultural University, Guangzhou, China

**Keywords:** *Pantoea ananatis*, bacterial wilt, plant-bacterium interactions, comparative genomics, host-contact metabolomics, host-associated phenotype

## Abstract

*Pantoea ananatis* is a widely distributed plant-associated bacterium that can exhibit contrasting ecological functions, ranging from plant-beneficial activities to pathogenicity. However, the genomic and metabolic features associated with such functional divergence among closely related strains remain poorly understood. Here, we characterized 18 mulberry-associated *P. ananatis* isolates using phenotypic assays and selected Pa-6 and YSFJ as representatives of two predominant functional types for comparative genomic and host-contact metabolomic analyses. Pa-6 exhibited a beneficial-associated phenotype characterized by several plant growth-promoting and antagonistic traits, comparatively limited tissue damage, and lower disease severity during the later stages of co-inoculation with *Ralstonia pseudosolanacearum* XZqk. In contrast, YSFJ caused progressive wilt accompanied by bacterial aggregation, xylem occlusion, vascular disorganization, and tissue damage. Despite these contrasting phenotypes, Pa-6 and YSFJ shared approximately 98.9% average nucleotide identity. Comparative genomic analyses revealed differences in accessory-gene content, plasmid composition, and strain-specific homologous clusters. Annotation comparisons highlighted a GH19-associated locus in Pa-6 and wbpZ- and feoB-associated loci in YSFJ as putative gene-content differences. Exploratory pathway mapping of host-contact extracellular metabolomic profiles highlighted central-carbon, organic-acid, and redox-related processes in the Pa-6 treatment and carbohydrate transport and utilization, aromatic-compound metabolism, and stress-related processes in the YSFJ treatment. These results document contrasting host-associated phenotypes and provide genomic and metabolic context for further investigation, while the contributions of individual loci and phylogenetic background remain unresolved.

## Graphical abstract

**Figure:**
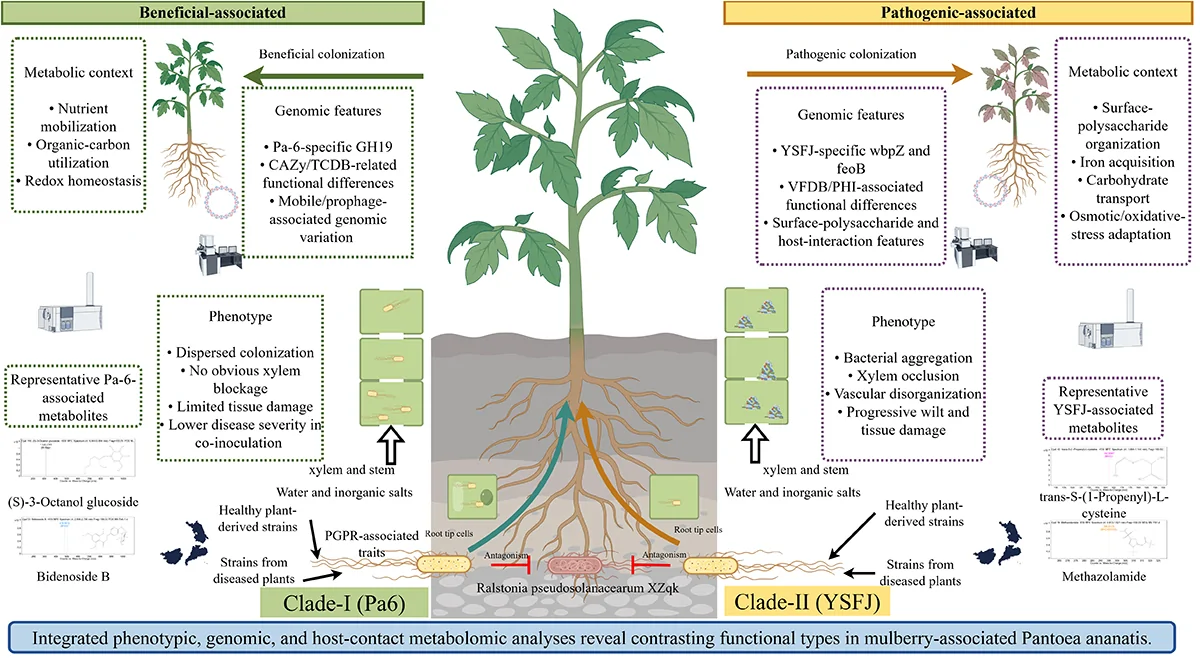
Contrasting host-associated phenotypes and candidate genomic–metabolic features of mulberry-associated *Pantoea ananatis*.

Integrated phenotypic, genomic, and host-contact metabolomic analyses distinguished two predominant functional types among mulberry-associated *P. ananatis*. Pa-6 represented the beneficial-associated type and was characterized by a comparatively scattered bacterial distribution in the examined tissues, no obvious xylem blockage, limited tissue damage, and lower disease severity during later stages of co-inoculation with *Ralstonia pseudosolanacearum* XZqk. In contrast, YSFJ represented the pathogenic-associated type and was associated with bacterial aggregation, xylem occlusion, vascular disorganization, progressive wilt, and tissue damage. Comparative genomic analyses identified strain-associated differences in gene-cluster representation and annotation, including putative differences involving GH19-, wbpZ-, and feoB-associated loci, together with differences in carbohydrate-active, transport, host-interaction, and mobile-genetic-element-related annotations. Host-contact metabolomic profiling further associated Pa-6 with nutrient mobilization, carbon utilization, and redox homeostasis, whereas YSFJ was associated with surface-polysaccharide functions, iron acquisition, carbohydrate transport, and stress adaptation. Representative treatment-associated metabolic features are shown for each inoculated condition. Both Pa-6 and YSFJ exhibited in vitro antagonistic activity against XZqk, despite their contrasting effects on mulberry. The illustrated genomic and metabolic features represent candidate functional contexts associated with the observed phenotypes rather than experimentally validated causal mechanisms. By Figdraw2.0 ID: WTUUR754c9.

## Introduction

*Pantoea ananatis* is a widely distributed plant-associated bacterium with considerable strain-level variation in its effects on plants. Members of this species have been recovered from soil, plant surfaces, internal tissues, and insect-associated environments, and individual strains may behave as pathogens, commensals, endophytes, or plant-beneficial bacteria depending on the host and environmental context [1–3]. Pathogenic strains have been associated with diseases of onion, maize, rice, eucalypt, and other crops, whereas other strains colonize plants without causing severe damage or express traits associated with plant growth promotion [3,4]. These contrasting effects can occur even among closely related isolates. Three maize-seed endophytes with highly similar genomic backgrounds showed growth-promoting, commensal, and weakly pathogenic phenotypes [5], while several *P. ananatis* isolates recovered from maize white-spot lesions failed to reproduce disease after reinoculation and retained plant growth-promoting characteristics [4]. Species identity or phylogenetic relatedness alone therefore provides only limited information about the eventual outcome of a *P. ananatis*–plant interaction.

This functional diversity is further complicated by the widespread occurrence of traits traditionally associated with plant growth promotion. Plant-associated *P. ananatis* strains can produce indole-3-acetic acid and siderophores, solubilize mineral nutrients, use ACC, and antagonize competing microorganisms [6–9]. A sugarcane-derived strain showed phosphate and zinc solubilization together with siderophore and IAA production and was able to colonize plants [7], while the rice endophyte *P. ananatis* D1 displayed phosphate-solubilizing, IAA-producing, ACC-deaminase, and siderophore activities and promoted rice growth under saline conditions [8]. *P. ananatis* has also been recovered among tomato root endophytes with antagonistic activity against *Ralstonia solanacearum* [9]. However, the presence of one or more of these traits does not necessarily predict whether a strain will be beneficial or damaging in planta [4,5]. Direct assessment of host responses is therefore needed alongside in vitro functional screening.

Comparative genomics offers a way to examine why closely related strains produce different host outcomes. *P. ananatis* has a relatively conserved core genome but a large accessory component that includes plasmids, mobile genetic elements, secretion-associated loci, and other variable regions linked to environmental adaptation and host interaction [2,5,10]. Onion-associated populations can show limited core-genome divergence despite marked variation in pathogenicity, with accessory-gene content more closely associated with phenotype [11]. Pan-genome-wide association analysis has likewise identified accessory loci associated with onion pathogenicity while showing that no single locus universally predicts pathogenic behavior [12]. Plasmid content may add another layer of variation; elimination of an endogenous plasmid from a pathogenic *P. ananatis* strain reduced disease severity and altered biofilm formation [13]. These observations point to combinations of accessory genes and other strain-specific genomic differences as more likely explanations for functional divergence than overall genomic similarity alone. Host-associated metabolic profiling may further help identify the physiological processes in which these genomic differences are expressed during plant interaction.

Mulberry is an economically important crop that supports sericulture and related industries in China [14]. *P. ananatis* has been implicated in mulberry bacterial diseases. Strain LCFJ-001 was isolated from bacterial-wilt-affected mulberry and reproduced wilt symptoms after inoculation [15], and subsequent surveys have detected *P. ananatis* among bacterial pathogens associated with mulberry wilt or bacterial blight [16,17]. Comparisons of healthy and diseased mulberry plants, as well as cultivars differing in wilt resistance, have also revealed diverse endophytic bacterial communities that include *Pantoea* and other taxa with potential roles in disease suppression [18,19]. Microscopic studies in other hosts provide additional evidence that *P. ananatis* can occur within diseased plant tissues; for example, bacterial cells have been visualized by transmission electron microscopy in maize white-spot lesions [20]. Despite these observations, the extent of functional variation among mulberry-associated *P. ananatis* isolates remains poorly resolved, particularly the relationship between phylogenetic position, plant-beneficial traits, pathogenicity, tissue-associated behavior, and strain-level genomic variation.

We therefore asked whether mulberry-associated *P. ananatis* isolates could be separated into reproducible functional types using an integrated set of phenotypic and host-interaction traits, and whether such differences could be related to strain-level genomic and metabolic variation. Specifically, we examined whether (i) plant growth-promoting, antagonistic, root-growth, and disease-associated phenotypes separated the isolates into contrasting groups and how these groups related to multilocus sequence phylogeny; (ii) representative isolates differed in their effects on whole plants and in their tissue-associated distribution, aggregation, vascular changes, and host damage; and (iii) these contrasting phenotypes were associated with differences in accessory-genome composition, strain-specific genomic features, and extracellular metabolic profiles under host-contact conditions.

Eighteen mulberry-associated *P. ananatis* isolates were first characterized by multilocus sequence analysis together with plant growth-promoting, antagonistic, root-growth, and branch-explant assays. Pa-6 and YSFJ were then selected as representatives of the contrasting phenotype-based groups and compared further using whole-plant inoculation, microscopic observation, complete-genome sequencing, comparative genomics, and host-contact metabolomic profiling. Genomic and metabolomic data were integrated at the pathway level to identify biological processes in which strain-associated genomic differences coincided with treatment-associated metabolic patterns and to prioritize candidate genes and pathways for subsequent functional validation.

## Methods

### Sampling and preparation of bacterial strains and plant materials

#### Strain sampling and identification

Eighteen *Pantoea ananatis* isolates were obtained from asymptomatic or symptomatic tissues of Morus atropurpurea and M. multicaulis collected in Guangdong, Guangxi, and Jiangsu Provinces between October 2018 and August 2023 (Table 1; Figure 1). Four isolates, listed as LCFJ-001, YSFJ, YDFJ2, and YDFJ6 in Table 1, were recovered from roots of symptomatic mulberry plants, whereas the remaining isolates were recovered from stems. Pa-6 was isolated from the stem of an asymptomatic plant, and YSFJ was isolated from the root of a plant showing whole-plant wilt and xylem blackening. The sampling design included isolates from contrasting symptom-associated sources and was not intended to evaluate geographical or climatic effects. *Ralstonia pseudosolanacearum* XZqk was included as the target bacterium in antagonism and co-inoculation assays.

**Figure 1.**
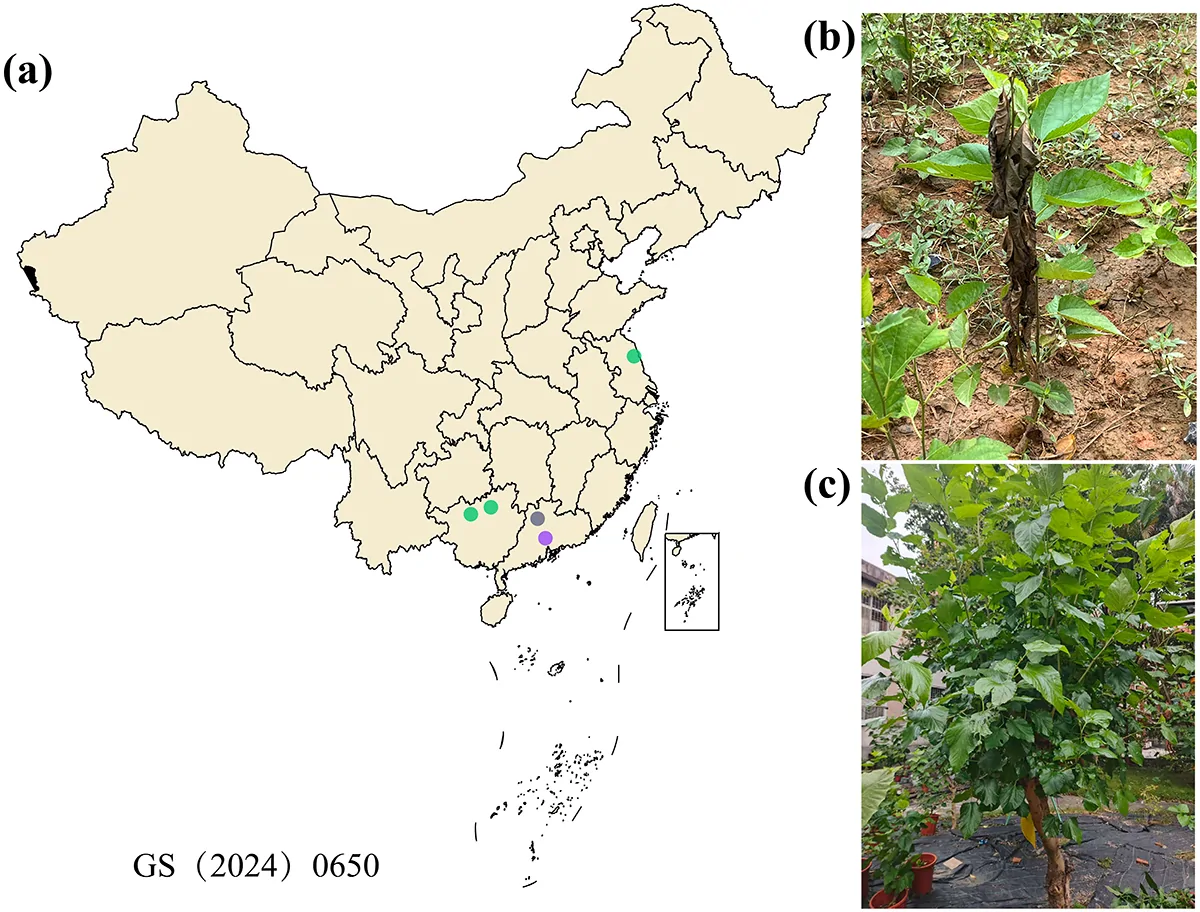
Sampling locations and representative symptom-associated source phenotypes of mulberry-associated *P. ananatis* isolates.

(a) Geographic distribution of the sampling locations in China. Sampling sites are shown according to the locations from which the mulberry-associated bacterial isolates were obtained. The sampling design was used to obtain isolates from contrasting symptom-associated source plants and was not intended to evaluate geographical or climatic effects.

(b) Representative symptomatic mulberry plant showing whole-plant wilting and vascular blackening at the Qingyuan sampling site (113.056°E, 23.686°N; May 2023).

(c) Representative asymptomatic mulberry plant at the Guangzhou sampling site (113.266°E, 23.131°N; March 2022).

**Table 1.** Information of strains used in this study.

Name	Strain Species	Latitude and longitude	Region	Host plant species	Source	Symptoms	Collection time	GDMCC
Pa-1	*P. ananatis*	113.266°E, 23.131°N	Guangdong Guangzhou	*M. atropurpurea* Sha2×Lun109	stem	Asymptomatic (Healthy Mulberry)	March 2022	–
Pa-2	*P. ananatis*	113.266°E, 23.131°N	Guangdong Guangzhou	*M. atropurpurea* Sha2×Lun109	stem	Asymptomatic (Healthy Mulberry)	March 2022	–
Pa-3	*P. ananatis*	113.266°E, 23.131°N	Guangdong Guangzhou	*M. atropurpurea* Sha2×Lun109	stem	Asymptomatic (Healthy Mulberry)	March 2022	–
Pa-4	*P. ananatis*	113.266°E, 23.131°N	Guangdong Guangzhou	*M. atropurpurea* Sha2×Lun109	stem	Asymptomatic (Healthy Mulberry)	March 2022	–
Pa-5	*P. ananatis*	113.266°E, 23.131°N	Guangdong Guangzhou	*M. atropurpurea* Sha2×Lun109	stem	Asymptomatic (Healthy Mulberry)	March 2022	–
Pa-6	*P. ananatis*	113.266°E, 23.131°N	Guangdong Guangzhou	*M. atropurpurea* Kang Qing10	stem	Asymptomatic (Healthy Mulberry)	March 2022	66,555
Pa-7	*P. ananatis*	113.266°E, 23.131°N	Guangdong Guangzhou	*M. atropurpurea* Kang Qing10	stem	Asymptomatic (Healthy Mulberry)	March 2022	–
Pa-8	*P. ananatis*	113.266°E, 23.131°N	Guangdong Guangzhou	*M. atropurpurea* Lun Jiao40	stem	Asymptomatic (Healthy Mulberry)	March 2022	–
Pa-9	*P. ananatis*	113.266°E, 23.131°N	Guangdong Guangzhou	*M. atropurpurea* Lun Jiao40	stem	Asymptomatic (Healthy Mulberry)	March 2022	–
Pa-10	*P. ananatis*	113.266°E, 23.131°N	Guangdong Guangzhou	*M. atropurpurea* Tang10	stem	Asymptomatic (Healthy Mulberry)	March 2022	–
Pa-11	*P. ananatis*	113.266°E, 23.131°N	Guangdong Guangzhou	*M. atropurpurea* Tang10	stem	Asymptomatic (Healthy Mulberry)	March 2022	–
Pa-12	*P. ananatis*	113.266°E, 23.131°N	Guangdong Guangzhou	*M. atropurpurea* Tang10	stem	Asymptomatic (Healthy Mulberry)	March 2022	–
LCFJ	*P. ananatis*	109.416°E, 24.325°N	Guangxi Liuzhou	*M. atropurpurea* Gui Sang6	root	The whole plant withers and the xylem turns black	October 2018	1.1601
YSFJ	*P. ananatis*	113.056°E, 23.686°N	Guangdong Qingyuan	*M. atropurpurea* Yue Sang 11	root	The whole plant withers and the xylem turns black	May 2023	–
YDFJ2	*P. ananatis*	113.056°E, 23.686°N	Guangdong Qingyuan	*M. atropurpurea* Yue Sang 11	root	The whole plant withers and the xylem turns black	May 2023	–
YDFJ6	*P. ananatis*	113.056°E, 23.686°N	Guangdong Qingyuan	*M. atropurpurea* Yue Sang 11	root	The whole plant withers and the xylem turns black	May 2023	–
YZFJ	*P. ananatis*	108.086°E, 24.693°N	Guangxi Hechi	*M. multicaulis Perr*. Nong Sang14	stem	The top of the plant turns black and withers	August 2023	–
JSFJ	*P. ananatis*	120.162°E, 33.348°N	Jiangsu Yancheng	*M. multicaulis Perr*. Xuan 792	stem	The top of the plant turns black and withers	August 2023	–
XZqk	*R. pseudosolanacearum*	109.416°E, 24.325°N	Guangxi Laibin	*M. atropurpurea* Gui Sang6	root	The whole plant withers and the xylem turns black	October 2021	1.1619

Bacterial isolates were obtained from surface-sterilized mulberry tissues and preliminarily identified by 16S rRNA gene sequencing followed by BLASTn comparison against the NCBI nucleotide database [21]. Detailed isolation and purification procedures are provided in Supplementary Methods S1. Pa-6 and LCFJ-001 were deposited in the Guangdong Microbial Culture Collection Center under accession numbers GDMCC 66,555 and GDMCC 1.1601, respectively.

#### Plant material

Seeds of the mulberry cultivar “Gui Sang 12” (*Morus atropurpurea*) were surface sterilized and germinated on Murashige and Skoog medium [22]. Sixty-day-old axenic seedlings were used for inoculation, microscopic observation, and metabolomic experiments, and only seedling batches showing no culturable bacterial contamination were used.

Rooted branch explants were prepared from 15-year-old “Lun Jiao 40” mulberry trees using a procedure adapted from Yuan et al. (2023b). Detailed procedures for seed sterilization, axenic-status screening, soil preparation, and branch-explant culture are provided in Supplementary Methods S1.

#### Nucleic acid extraction

Genomic DNA extracted from pure bacterial cultures was used for amplification and sequencing of the 16S and 23S rRNA genes, *gyrB*, *leuS*, *pleD*, and *rpoB*. Detailed bacterial culture and DNA-extraction procedures are provided in Supplementary Methods S1.

### Multilocus sequence analysis

Partial sequences of the 16S and 23S rRNA genes and the protein-coding loci *gyrB*, *leuS*, *pleD*, and *rpoB* were amplified and sequenced from all 18 *P. ananatis* isolates. Primer sequences and locus-specific information are provided in Table 2. The 16S and 23S rRNA primers were derived from Jagoueix et al. [23] and Liu et al. [24], Chinese Patent CN120118770A], respectively; primers for *pleD*, *leuS*, and *rpoB* were reported by Yuan et al. [16] and Sun et al. [25]. Selection of the protein-coding loci was additionally guided by previous multilocus sequence analyses of *Pantoea* and *P. ananatis* populations [11,27,28].

**Table 2.** Primer information used in this study.

Gene Name	Primer Name	Sequence (5”−3”)	Primer Length (bp)	Amplification Length (bp)	Reference
*pleD*()	PleDF	CATCTACGAACGCTCCTTACCGC	23	929	Yuan et al. [16]
	PleDR	CGCACCATTCCGGCCAGTTT	20		
*16S rRNA*	27-F	AGAGTTTGATCATGGCTCAG	20	1542	Jagoueix et al. [23]
	1492-R	AAGGAGGTGATCCAGCCGC	20		
*23S rRNA*	23S-F	TGGAGGTATCAGAAGTGCGAAT	22	727	Liu et al. [24], CN120118770A
	23S-R	GGAACTTACCCGACAAGGAAT	21		
*rpoB*	rpoB-F	GGCTGAAAACCAGTTCCGCGT	21	846	Sun et al. [25]
	rpoB-R	CCAGAGAGACACACGGCATCT	21		
*leuS*	leuS-F	CAGTGGTTTATCAAAATCACC	21	726	Sun et al. [25]
	leuS-R	GGATAACCGGAAGCTGATCTT	21		
*gyrB*	gyrB-F	TACGGGAGATACCGATGC	18	692	Xiao et al. [26]
	gyrB-R	GCGTGCGGCGTCAATAAT	18		

PCR products were bidirectionally sequenced by Sanger sequencing, assembled and edited using BioEdit [29], and aligned separately by locus using ClustalW [30]. The six loci were trimmed to common alignment lengths and concatenated in the order 16S rRNA–23S rRNA–*gyrB*–*leuS*–*pleD*–*rpoB*. Detailed PCR, sequencing, and sequence-processing procedures are provided in Supplementary Methods S2.

The MLSA dataset comprised the 18 mulberry-derived isolates and nine publicly available *P. ananatis* reference strains selected to provide broader phylogenetic context (Table S1). A maximum-likelihood tree was reconstructed in MEGA 12 under the GTR+G substitution model with 1,000 bootstrap replicates [31]. MLSA clades were assigned descriptively according to major groupings in the resulting tree topology; no separate clustering algorithm or predefined genetic-distance threshold was used to define the clades.

### Functional assays of plant growth-promoting and antagonistic traits

Seven PGPR-associated traits were evaluated for all 18 *P. ananatis* isolates: growth under nitrogen-free conditions, phosphate and potassium solubilization, ACC utilization, indole-3-acetic acid production, siderophore production, and cellulase activity. These assays represented complementary functions related to nutrient acquisition and mobilization, phytohormone and stress modulation, iron acquisition, and plant-associated substrate utilization. Nitrogen fixation-related growth, phosphate and potassium solubilization, siderophore production, ACC utilization, indole-3-acetic acid production, and cellulase activity were assessed using established methods or assay principles [32–37]. Protease, β-1,3-glucanase, and hemolytic activities were additionally examined, with β-1,3-glucanase activity assessed following Walsh et al. [38].

Antagonistic activity against *R. pseudosolanacearum* XZqk was evaluated for all isolates using a plate-based inhibition assay. Quantitative measurements from the functional and antagonistic assays were subsequently integrated with root-growth and branch-lesion measurements for phenotype-based clustering (Integrated phenotypic clustering and visualization). Assay conditions, calculation procedures, and criteria used for categorical visualization in Figure 2(a) are provided in Supplementary Methods S3.

**Figure 2.**
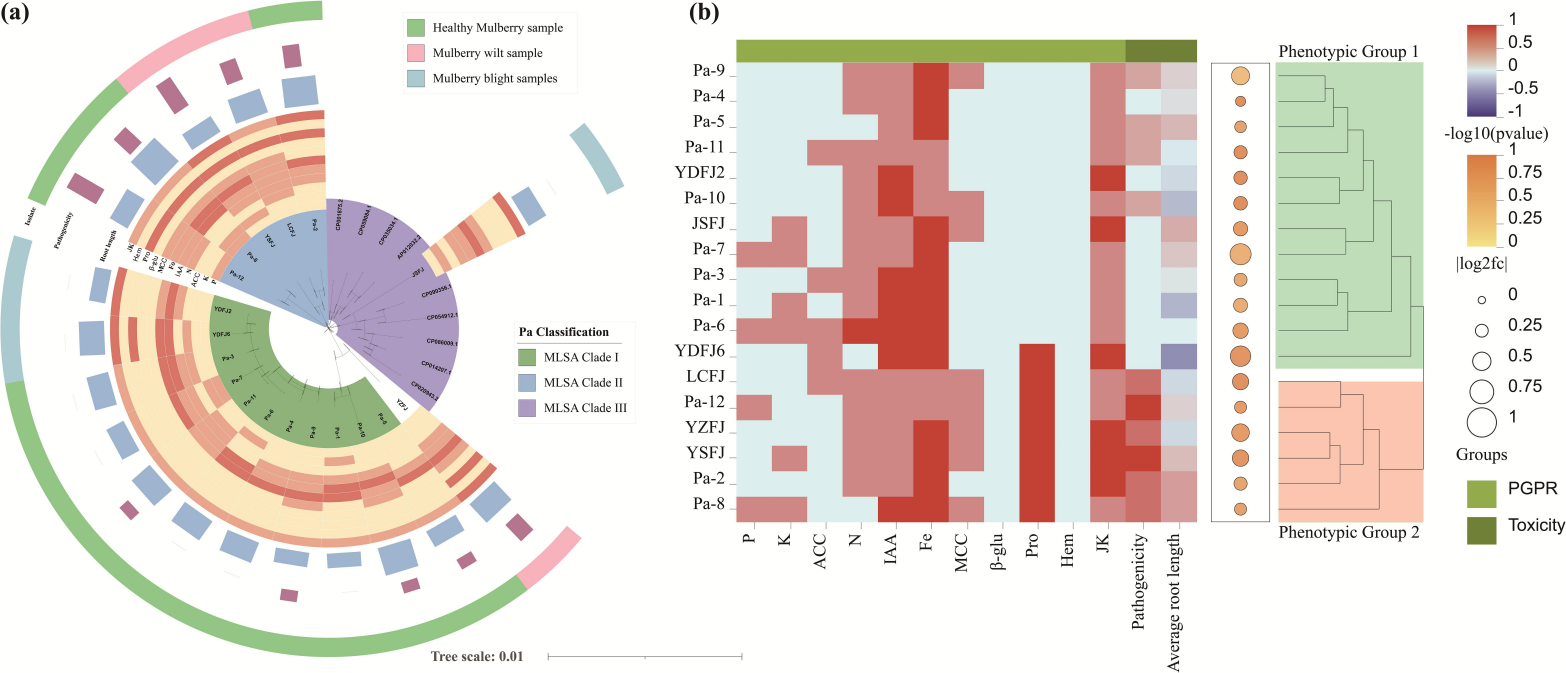
Molecular and phenotypic classification of mulberry-associated *Pantoea ananatis* isolates.

(a) Multilocus sequence analysis based on concatenated 16S rRNA, 23S rRNA, *gyrB*, *leuS*, *pleD*, and *rpoB* sequences. The tree includes 18 isolates obtained in this study and nine reference strains retrieved from public databases. Among the study isolates, MLSA Clade I contained 11 isolates, Clade II contained five isolates, and Clade III contained JSFJ, whereas YZFJ occupied an intermediate position between Clades I and III. All nine reference strains clustered within Clade III. From the outermost to the innermost tracks, the annotations indicate isolate source, pathogenicity, root-length response, antagonistic activity against *R. pseudosolanacearum* XZqk, and plant growth-promoting traits. Isolate source is indicated by green for isolates from asymptomatic mulberry plants, red for isolates from mulberry plants showing wilt symptoms, and blue for isolates from mulberry plants showing blight symptoms. Pathogenicity and root-length responses are represented by bars, with longer bars indicating greater pathogenicity and stronger root-growth promotion, respectively. For JK, Hem, Pro, β-glu, MCC, Fe, IAA, N, ACC, K, and P, yellow indicates no detectable activity or utilization, pink indicates intermediate activity or utilization, and red indicates strong activity or utilization.

(b) Phenotype-based hierarchical clustering of the 18 mulberry-derived isolates using PGPR-associated traits, antagonistic activity, and plant-interaction measurements. Trait values were Z-score standardized before clustering. Pairwise Euclidean distances were calculated from the Z-score-standardized trait matrix, followed by complete-linkage hierarchical clustering. The dendrogram represents phenotypic similarity among isolates and does not indicate statistical correlations between individual traits. Colored points represent individual isolates; yellow points indicate isolates displayed in the two-dimensional visualization and do not represent an additional biological category.

### Root-growth, branch-explant, and integrated phenotypic analyses

#### Root-growth assay

The effects of the 18 *P. ananatis* isolates on mulberry root growth were evaluated using surface-sterilized seeds of “Gui Sang 12.” The assay was adapted from previous approaches used to evaluate *P. ananatis*-mediated effects on seedling and root development [4,8]. Seeds were inoculated with bacterial suspensions adjusted to an OD_600_ of 1.0, and primary root length was measured after 7 d. Each assay was conducted in triplicate. Detailed inoculation and culture procedures are provided in Supplementary Methods S4.

#### Branch-explant assay

The effects of the 18 *P. ananatis* isolates on mulberry branch tissues were evaluated using rooted “Lun Jiao 40” explants. The assay was adapted from the mulberry branch inoculation system described by Yuan et al. (2023b). Explants were inoculated by stem puncture with bacterial suspensions adjusted to an OD_600_ of 0.1, and lesion development was evaluated at 7 d post-inoculation. Each assay was conducted in triplicate. Bacteria were re-isolated from symptomatic tissues to assess consistency with the corresponding inoculated isolates. Detailed inoculation and assessment procedures are provided in Supplementary Methods S4.

#### Integrated phenotypic clustering and visualization

Quantitative functional and antagonistic traits measured in Functional assays of plant growth-promoting and antagonistic traits were integrated with root-length and branch-lesion measurements for the 18 isolates. Each trait was standardized by Z-score transformation, followed by hierarchical clustering using Euclidean distance and complete-linkage agglomeration in CNSKnowall. Functional groups were defined from the integrated phenotypic profiles rather than from isolate origin, individual traits, or MLSA phylogenetic position.

The standardized isolate-by-trait matrix was additionally displayed using the CNSKnowall bubble-heatmap module. This visualization was descriptive and did not independently contribute to definition of the phenotype-based groups.

### Representative-strain antagonism validation and whole-plant inoculation assay

Pa-6 and YSFJ were selected as representative isolates of the two contrasting phenotype-based functional groups identified in Integrated phenotypic clustering and visualization. *R. pseudosolanacearum* XZqk was used as the target pathogen. Antagonistic activity of Pa-6 and YSFJ culture supernatants against XZqk was evaluated using an agar-well diffusion assay.

For whole-plant validation, six treatments were established: CK, Pa-6, Pa-6 + XZqk, YSFJ, YSFJ + XZqk, and XZqk, with 15 independent plants per treatment. Bacterial suspensions at approximately 10^8^ CFU mL^−1^ were applied by root drenching after standardized root wounding. Disease severity was assessed at 1, 3, 6, 9, 12, and 15 d post-inoculation using a 0–5 ordinal disease-rating system adapted from Yuan et al. [16].

Pairwise treatment comparisons were performed independently at each observation time using two-sided Wilcoxon rank-sum tests, with *p* values adjusted by the Holm method. Differences were considered significant at Holm-adjusted *p* < 0.05. Detailed antagonism, inoculation, disease-scoring, and visualization procedures are provided in Supplementary Methods S5.

### Scanning electron microscopy of bacterial cell morphology

Cell morphology of purified bacterial isolates was examined by scanning electron microscopy using a Hitachi S-3400N instrument. Samples were prepared using standard fixation, dehydration, critical-point drying, and metal-coating procedures for biological specimens [39,40]. Detailed sample-preparation and imaging conditions are provided in Supplementary Methods S6.

### Temporal symptom assessment and microscopic observation of colonization in axenic mulberry seedlings

Sixty-day-old axenic “Gui Sang 12” mulberry seedlings were inoculated with Pa-6 or YSFJ using a root-contact method adapted from previously described axenic mulberry inoculation systems (Yuan et al., 2023a [16];). Uninoculated seedlings served as controls. Visible symptoms and root and stem tissues were examined before inoculation and at 1, 3, 5, 10, and 15 d post-inoculation.

At each sampling time, three independent seedlings were examined per treatment. For each microscopy method, 10 microscopic fields distributed across the three biological replicates were evaluated qualitatively for bacterial presence and distribution, aggregation, starch-granule appearance, vascular organization, xylem occlusion, and cellular or tissue damage.

Root and stem tissues were examined using scanning electron microscopy, transmission electron microscopy, and paraffin-section light microscopy following established biological microscopy and plant histology procedures [39–42]. Detailed inoculation, tissue preparation, sectioning, staining, and imaging procedures are provided in Supplementary Methods S7.

### Genome sequencing, annotation, and comparative genomic analyses

Pa-6 and YSFJ were selected for complete-genome sequencing and comparative genomic analysis. The comparative dataset comprised 41 *Pantoea ananatis* genomes: 38 publicly available reference genomes, the previously published mulberry-associated strain LCFJ-001, and Pa-6 and YSFJ sequenced in the present study. Reference genomes were selected through a literature-guided search to represent diverse host or isolation sources and previously reported ecological or functional backgrounds. Genome assemblies were retrieved from the NCBI Assembly database on 12 October 2025. Assembly accessions, host or isolation sources, geographical origins, literature-based functional or ecological categories, and supporting references are provided in Table S7. The dataset was used to provide phylogenomic and gene-content context for Pa-6 and YSFJ rather than as an exhaustive survey of publicly available *P. ananatis* genomes.

#### DNA extraction, sequencing, quality control, and genome assembly

Pa-6 and YSFJ were sequenced using complementary short- and long-read sequencing. Short reads were quality filtered with Trimmomatic v0.36 [43], and long reads were assembled de novo using Canu v1.3 [44]. Residual gaps and sequence errors were corrected using GapFiller, PrInSeS-G, and short-read polishing with Pilon [45–47].

Final chromosome and plasmid assemblies were evaluated for circularity, sequencing coverage, and assembly quality. Genome completeness was assessed using BUSCO [48], while completeness and contamination were independently evaluated using CheckM [49]. Circular genome maps were generated using CGView [50]. Detailed DNA-quality assessment, assembly processing, circularization, polishing, and genome-quality evaluation procedures are provided in Supplementary Methods S8.1.

#### Structural and functional genome annotation

Structural annotation of the Pa-6 and YSFJ genomes was performed using the NCBI Prokaryotic Genome Annotation Pipeline (PGAP), and Prokka was additionally used to generate standardized GFF files for downstream comparative analyses [51,52].

Predicted proteins were functionally annotated using general sequence-, domain-, orthology-, and pathway-based resources, including NR, COG, CDD, Pfam, GO, and KEGG [21,53–59]. Specialized annotations included virulence- and pathogen–host interaction-associated proteins, antimicrobial and metal-resistance-associated proteins, transporters, carbohydrate-active enzymes, membrane and secretion-related features, genomic islands, prophages, and biosynthetic gene clusters [60–73].

Sequence-similarity-based functional assignments were treated as annotations rather than experimental confirmation of the corresponding biological functions. Detailed database versions, search tools, thresholds, and prediction procedures are provided in Supplementary Methods S8.2.

Plasmid-encoded CDSs were additionally examined by replicon, and their existing PHI-base, VFDB, CARD, and SARG annotations were used to assess plasmid-associated host-interaction- and resistance-related sequence matches (Tables S4–S5).

#### Comparative overlap of annotation and prediction identifiers

Nonredundant annotation or prediction identifiers from the principal functional databases were compared between Pa-6 and YSFJ as a complementary functional comparison. These analyses were performed at the annotation-ID level and were not interpreted as direct evidence of gene-content differences. Detailed procedures are provided in Supplementary Methods S8.3, with corresponding results in Figure S4 and Tables S4–S5.

#### Average nucleotide identity and phylogenomic analyses

Pairwise average nucleotide identity (ANI) was calculated among the 41 *P. ananatis* genomes using fastANI. Genome pairs without an available ANI estimate were retained as missing values rather than assigned an arbitrary similarity value.

Genome-derived 16S rRNA and single-copy core-gene phylogenies were reconstructed using a subset of 21 genomes. The 16S rRNA sequences were aligned using MAFFT and analyzed using FastTree [74,75]. For the core-gene phylogeny, single-copy core-gene sequences were concatenated, aligned using MAFFT, and analyzed using FastTree [52,75,76].

Genome-wide SNP analysis was performed across the 41-genome dataset using the LCFJ-001 chromosome (CP066803) as the common reference with Snippy v4.6.0. Individual outputs were combined using snippy-core to generate the core-SNP alignment, which was used for phylogenetic reconstruction with FastTree.

Available host or isolation source, functional category, geographical origin, and other strain metadata were mapped onto the resulting trees for descriptive comparison. Geographic information was standardized at the continental level, and annotated trees were visualized using *ggtree* [77]. Detailed sequence extraction, alignment, clustering, SNP-processing, and visualization procedures are provided in Supplementary Methods S8.4.

#### Pan-genome and gene-content analyses

Pan-genome analysis of the 41 *P. ananatis* genomes was performed using Roary with standardized Prokka-generated GFF files [76]. Homologous gene clusters were classified as core, shell, or cloud according to their distribution across the analyzed genomes, and strain-specific clusters were defined as clusters detected in only one genome.

Core- and pan-genome diversity, functional-category distributions, and genome-wide gene presence–absence patterns were further summarized from the Roary output, with COG classifications assigned at the homologous-cluster level [78]. Detailed rarefaction, COG-classification, and presence–absence visualization procedures are provided in Supplementary Methods S8.5.

To distinguish pairwise differences from strain specificity across the full dataset, the Pa-6 and YSFJ columns of the Roary-derived copy-number matrix were compared directly. A cluster was considered present when its recorded copy number was greater than zero. Clusters were classified as shared by both strains, detected in Pa-6 but not YSFJ, or detected in YSFJ but not Pa-6. Copy-number differences were also summarized among shared clusters. These categories describe representation within the existing clustering framework and do not, by themselves, establish the absence of all homologous sequences from the other genome.

#### Pairwise whole-genome collinearity analysis

Pairwise whole-genome collinearity between Pa-6 and YSFJ was analyzed using MUMmer4, with Pa-6 as the reference assembly and YSFJ as the query assembly [79]. Collinear alignment blocks were summarized according to nucleotide-identity intervals, and their distributions were visualized using the R package *circlize* [80]. Detailed alignment processing, identity classification, and block-frequency calculations are provided in Supplementary Methods S8.6.

### Antibiotic inhibition-zone assay and resistance-associated genomic annotation

Antibiotic responses were examined as a supplementary comparison of strain-level variation in resistance- and stress-associated traits among the 18 mulberry-derived *P. ananatis* isolates. The isolates were evaluated using a disk-diffusion assay [81,82] with antimicrobial susceptibility disks (Hangzhou Binhe Microbial Reagent Co., Ltd., Hangzhou, China). Thirty-six antibiotics were tested, including β-lactams [cephalexin, cefuroxime, cephalothin, cefaclor, cefamandole, cefotaxime, ceftriaxone, ceftazidime, cefepime, ampicillin, amoxicillin, carbenicillin, mezlocillin, piperacillin, cefoxitin, cefmetazole, imipenem, meropenem, and aztreonam], aminoglycosides (streptomycin, kanamycin, amikacin, gentamicin, and tobramycin), tetracyclines (minocycline, tetracycline, and doxycycline), quinolones (norfloxacin, ofloxacin, and ciprofloxacin), chloramphenicol, erythromycin, spectinomycin, polymyxin, nitrofurantoin, and trimethoprim–sulfamethoxazole.

Resistance-associated proteins in Pa-6 and YSFJ were additionally annotated against CARD and SARG. These analyses were used descriptively and were not used to define the phenotype-based functional groups. Detailed assay conditions, annotation procedures, and classification criteria are provided in Supplementary Methods S9.

### Untargeted metabolomics and KEGG pathway-level co-mapping

Sterile 60-day-old “Gui Sang 12” mulberry seedlings were assigned to CK, Pa-6, and YSFJ treatments, with three independent biological replicates per treatment and three technical LC–MS injections per biological replicate. Culture medium surrounding the seedlings was collected at 15 d post-inoculation, clarified and filtered, and subjected to untargeted metabolomic analysis using an Agilent 6545B Q-TOF LC/MS system in both positive- and negative-ionization modes.

Raw LC–MS data were processed using Agilent MassHunter Qualitative and Quantitative Analysis v10.0. Technical injections from each biological replicate were averaged before statistical analysis, consistent with established metabolomics reporting principles [83,84]. The resulting abundance matrix was processed in MetaboAnalyst 5.0 using sum normalization, log_2_ transformation, and autoscaling [85]. Features consistently detected in all three CK biological replicates were removed before comparison of the Pa-6- and YSFJ-inoculated treatments. Detailed sample preparation, chromatographic and mass-spectrometric conditions, feature-extraction parameters, and preprocessing procedures are provided in Supplementary Methods S10.1.

Univariate comparisons between the Pa-6- and YSFJ-inoculated treatments were performed using feature-specific two-sided independent-samples *t*-tests or Wilcoxon rank-sum tests, with *p* values adjusted using the Benjamini–Hochberg false discovery rate procedure [86]. Features were assigned to Pa-6- or YSFJ-associated sets according to the direction of the mean normalized abundance difference, independently of statistical significance. Detailed feature-level test assignments are provided in Supplementary Methods S10.2 and Table S11.

Metabolites were putatively annotated using accurate-mass matching and, where available, MS/MS information against METLIN, with PlantCyc used for cross-reference [87,88]. Because authentic standards were not available for all detected features, annotations were treated as putative identifications [83,84]. Annotated features were mapped to KEGG compound identifiers for downstream pathway analysis [58,89].

Because a *P. ananatis*-specific pathway library was not available in MetaboAnalyst, the *Escherichia coli* K-12 MG1655 pathway library was used as an available bacterial reference framework. This library was used only for pathway mapping and was not used to infer the biological origin of detected metabolites. Pathway over-representation was evaluated using the hypergeometric test and pathway topology using relative-betweenness centrality [85,90].

Strain-specific homologous gene clusters identified by Roary were assigned KEGG Orthology identifiers, and pathways represented by these KOs were compared with those represented by treatment-associated KEGG compounds [76,89]. Pathways represented in both datasets were retained for pathway-level co-mapping. These associations were interpreted strictly as pathway co-membership and not as evidence of direct gene–metabolite regulation.

A normalized pathway mapping score was used to account for differences in the total numbers of pathway-mapped entries between the genomic and metabolomic datasets. Pathway-level networks were visualized using Cytoscape v3.9.1 [91]. Detailed compound mapping, pathway scoring, and network-construction procedures are provided in Supplementary Methods S10.2.

### Statistical analyses and figure preparation

Computational analyses were performed using the software and procedures specified in the corresponding sections. Available analysis inputs, outputs, recorded parameters, and reproducibility information are provided in the public repository described in the Data Availability section and in Supplementary Methods S11.

## Results

### *Molecular and phenotypic classification of mulberry-associated* P. ananatis *isolates*

The 18 mulberry-associated isolates were obtained from geographically distinct sampling sites in Guangdong, Guangxi, and Jiangsu Provinces (Figure 1(a)), including plants with disease-associated symptoms and asymptomatic plants, represented in Figure 1(b and 1), respectively. Scanning electron microscopy showed a broadly consistent rod-shaped cell morphology among the isolates (Figure S1), and multilocus sequence analysis supported their identification as *P. ananatis*.

MLSA resolved the study isolates into three principal phylogenetic groupings, with YZFJ occupying an intermediate position between two of the major clades (Figure 2(a)). The nine publicly available reference strains included for phylogenetic context clustered within Clade III.

The isolates showed substantial variation in plant-associated functional traits, root-growth responses, antagonistic activity, and branch-lesion phenotypes. Integration of these measurements separated the 18 isolates into two major phenotype-based groups (Figure 2(b)). Phenotypic Group 1 contained 12 isolates and was generally associated with stronger plant growth-promoting and antagonistic traits together with lower disease-associated measurements, whereas Phenotypic Group 2 contained six isolates and showed comparatively stronger disease-associated effects.

The phenotype-based grouping broadly, but not completely, corresponded to the MLSA topology (Figure 2(a,b)). Pa-6 and YSFJ were selected as representatives of the two contrasting phenotype-based groups for subsequent whole-plant, microscopic, genomic, and metabolomic comparisons. Detailed isolate-level phylogenetic positions and functional-trait patterns are provided in Supplementary Results SR1.

### In vitro antagonism and contrasting disease progression of Pa-6 and YSFJ in potted mulberry plants

Culture supernatants from both Pa-6 and YSFJ produced visible inhibition zones against *R. pseudosolanacearum* XZqk under the tested in vitro conditions (Figure 3(c and 3) insets). Despite this shared in vitro antagonistic activity, the two *P. ananatis* strains showed markedly different effects on potted mulberry plants.

**Figure 3.**
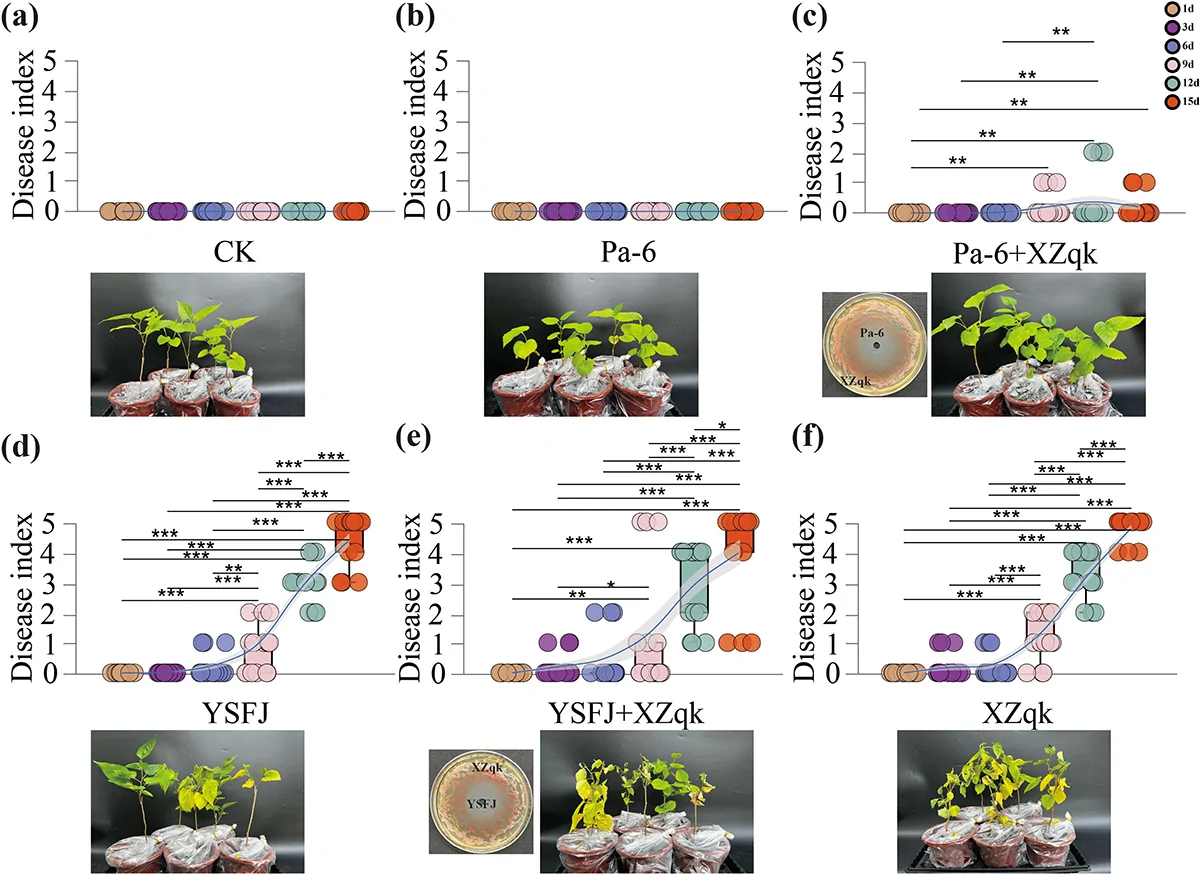
In vitro antagonism and disease progression following inoculation of potted mulberry plants with *Pantoea ananatis* pa-6, YSFJ, and *R. pseudosolanacearum* XZqk.

(a) Sterile-water control (CK).

(b) Plants inoculated with *P. ananatis* Pa-6 alone.

(c) Plants co-inoculated with Pa-6 and *R. pseudosolanacearum* XZqk at a 1:1 inoculum ratio; the inset shows the inhibition zone produced by the 72-h Pa-6 fermentation filtrate against XZqk on an agar plate.

(d) Plants inoculated with *P. ananatis* YSFJ alone.

(e) Plants co-inoculated with YSFJ and *R. pseudosolanacearum* XZqk at a 1:1 inoculum ratio; the inset shows the inhibition zone produced by the 72-h YSFJ fermentation filtrate against XZqk on an agar plate.

(f) Plants inoculated with *R. pseudosolanacearum* XZqk alone.

Potted-plant photographs were obtained at 15 days post-inoculation. The combined scatter and ribbon plot shows disease severity scores on a 0–5 ordinal scale at 1, 3, 6, 9, 12, and 15 days post-inoculation. Each treatment comprised 15 independent plants. Individual points represent the disease severity scores of individual plants, the central lines represent the mean scores, and the upper and lower boundaries of the shaded ribbons represent the mean ± standard error of the mean (SE). Pairwise comparisons among treatments were performed independently at each observation time using two-sided Wilcoxon rank-sum tests, with Holm adjustment for multiple comparisons within each time point. *Holm-adjusted *p < 0.05*, **Holm-adjusted *p* < 0.01, and ****Holm-adjusted *p* < 0.001; ns, not significant.

CK plants remained asymptomatic throughout the 15-d observation period (Figure 3(a)), as did plants inoculated with Pa-6 alone (Figure 3(b)). Plants co-inoculated with Pa-6 and XZqk developed only mild disease, and disease severity from 9 dpi onward remained significantly lower than that of plants inoculated with XZqk alone (Holm-adjusted *p* < 0.05; Figure 3 (and 3)).

In contrast, YSFJ alone caused progressive disease and severe wilting by 15 dpi (Figure 3), while YSFJ + XZqk also resulted in marked disease development (Figure 3(e)). At 15 dpi, YSFJ, YSFJ + XZqk, and XZqk showed significantly higher disease severity than CK, Pa-6, and Pa-6 + XZqk (Holm-adjusted *p* < 0.05). Detailed temporal disease-severity values and pairwise comparisons are provided in Supplementary Results SR2.

### Temporal symptom development and microscopic localization of Pa-6 and YSFJ in axenic mulberry seedlings

Uninoculated control seedlings remained asymptomatic throughout the experiment and showed regularly organized vascular tissues, clear xylem lumina, and starch-rich root-cap cells in the examined fields (Figure 4(a,d)).

**Figure 4.**
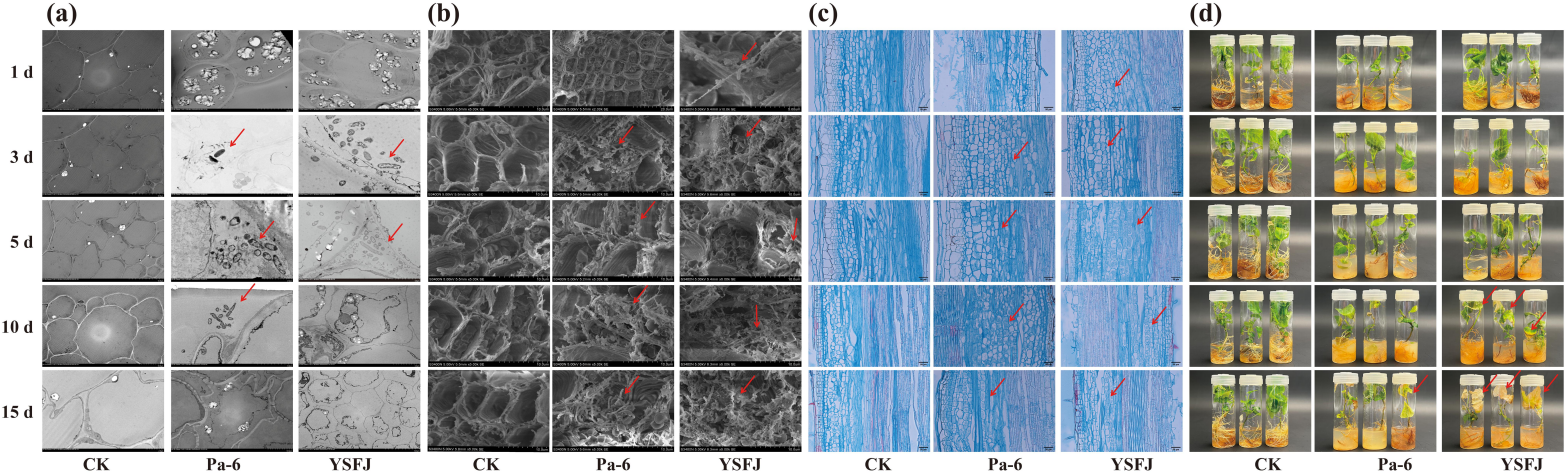
Temporal symptom development and microscopic tissue-associated changes in mulberry seedlings inoculated with *Pantoea ananatis* pa-6 or YSFJ.

(a) Transmission electron microscopy images of root-cap cells from uninoculated control, Pa-6-inoculated, and YSFJ-inoculated seedlings at 1, 3, 5, 10, and 15 days post-inoculation (dpi). The images show bacterial cells, cellular integrity, and changes in the visible abundance of starch granules. Magnification: 1,000×-2,000×.

(b) Scanning electron microscopy images of taproot xylem from control, Pa-6-inoculated, and YSFJ-inoculated seedlings at 1, 3, 5, 10, and 15 dpi. The images show bacterial distribution and aggregation, filamentous material, and vascular occlusion. Magnification: 5,000×-10000×.

(c) Paraffin sections of stem vascular tissues from control, Pa-6-inoculated, and YSFJ-inoculated seedlings at 1, 3, 5, 10, and 15 dpi. The sections show changes in vascular organization, xylem lumina, and tissue integrity. Magnification: 20 × .

(d) Visible symptoms of uninoculated control, Pa-6-inoculated, and YSFJ-inoculated seedlings at 1, 3, 5, 10, and 15 dpi. CK denotes the uninoculated axenic mulberry cultivar “Guisang 12” control.

Representative observations were obtained at 1, 3, 5, 10, and 15 days post-inoculation (dpi) from uninoculated controls and seedlings inoculated with Pa-6 or YSFJ. Transmission electron microscopy, scanning electron microscopy, histological sections, and whole-seedling phenotypes were used to visualize temporal changes in bacterial distribution and host tissue structure. Red arrows indicate bacterial cells observed in tissues from the inoculated treatments. Three independently inoculated seedlings were examined per treatment at each time point, with 10 microscopic fields evaluated across the three seedlings. Microscopy was used qualitatively to characterize tissue-associated bacterial distribution, aggregation, vascular changes, and tissue damage rather than to quantify total bacterial abundance; therefore, the number of bacterial cells visible in an individual field should not be interpreted as a quantitative measure of whole-seedling colonization.

YSFJ inoculation was associated with progressive tissue and whole-seedling changes. In the examined root-cap tissues, bacterial cells and aggregates became more evident at later observation times together with cellular disruption (Figure 4(a)), while scanning electron microscopy showed bacterial aggregation and progressive occlusion of taproot xylem lumina (Figure 4(b)). Paraffin sections showed increasing vascular disorganization and tissue damage at later observation times (Figure 4). These microscopic changes accompanied progressive leaf and stem yellowing, taproot blackening, and ultimately severe wilting by 15 dpi (Figure 4).

In contrast, bacterial cells observed in Pa-6-inoculated tissues generally showed a more scattered distribution, with no obvious vascular blockage in the examined fields (Figure 4(a,c)). Pa-6-inoculated seedlings remained asymptomatic during the earlier observation period and developed only mild yellowing at later time points (Figure 4). Thus, the two inoculation treatments showed clearly different temporal patterns of tissue association and host damage. Detailed observations at individual sampling times are provided in Supplementary Results SR3.

### *Whole-genome sequencing and comparative genomic analyses of two mulberry-derived* P. ananatis *isolates*

#### Genome assembly and quality assessment

Complete circular genome assemblies were obtained for both Pa-6 and YSFJ. The Pa-6 genome comprised a 4,700,721-bp chromosome and one 362,399-bp plasmid, resulting in a total genome size of 5,063,120 bp. The YSFJ genome comprised a 4,597,325-bp chromosome and two plasmids of 331,567 and 211,717 bp, resulting in a total genome size of 5,140,609 bp. Thus, YSFJ had a genome 77,489 bp larger than that of Pa-6 and contained one additional plasmid (Table S3; Figure S2(a–e)).

Both assemblies showed 99.98% genome coverage and 100% completeness according to CheckM and BUSCO. CheckM contamination estimates were 1.402% for Pa-6 and 1.1% for YSFJ. Sequencing-quality, depth, GC-distribution, assembly-quality, and circular-genome characteristics are summarized in Figure S2(a–e), with detailed sequencing and assembly statistics provided in Tables S2–S3 and Supplementary Results SR4.1.

Examination of the existing plasmid annotations showed host-interaction- and resistance-associated sequence matches on plasmids from both strains (Tables S4–S5). The additional 211,717-bp YSFJ plasmid contained sequences annotated with functions related to chemotaxis, fimbrial or adhesion-associated processes, autotransport, and membrane transport, together with a limited number of resistance-associated matches. T6SS-associated genes were detected on the principal plasmids of both Pa-6 and YSFJ. The candidate loci GH19, *wbpZ*, and *feoB* were not identified among the plasmid-encoded CDSs.

#### Gene prediction and functional annotation

The annotations summarized in Tables S4 and S5 contained 4,567 coding sequences (CDSs) for Pa-6 and 4,729 for YSFJ, a net difference of 162 CDSs. Pa-6 contained 4,233 chromosome-associated and 334 plasmid-associated CDSs, whereas YSFJ contained 4,208 chromosome-associated and 521 plasmid-associated CDSs across its two plasmids. In these annotation datasets, the higher total CDS count in YSFJ therefore reflected 187 more plasmid-associated CDSs and 25 fewer chromosome-associated CDSs. At least one functional annotation was assigned to 4,555 Pa-6 CDSs (99.74%) and 4,717 YSFJ CDSs (99.75%) (Table 3).

**Table 3.** Gene prediction and functional annotation statistics.

Feature Type	Pa-6		YSFJ	
	Gene number	Base number	Gene number	Base number
CDS	4567	4,285,689 bp	4729	4,433,412 bp
rRNA	22	32,056 bp	22	31,971 bp
tRNA	78	6105 bp	84	6620 bp
pseudo-gene	93	82,655 bp	0	0 bp
ncRNA	22	3323 bp	1	363 bp
Feature	4782	4,409,828 bp	4836	4,472,366 bp
Database	Number of genes	Percentage(%)	Number of genes	Percentage(%)
Annotated in NR	4554	99.72	4717	99.75
Annotated in COG	3392	74.27	3447	72.89
Annotated in CDD	2009	43.99	2111	44.64
Annotated in PFAM	3253	71.23	3298	69.74
Annotated in GO	2122	46.46	2146	45.38
Annotated in KEGG	1554	34.03	1592	33.66
Annotated in CAzy	86	1.88	88	1.86
Annotated in TCDB	704	15.41	723	15.29
Annotated in PHI	911	19.95	943	19.94
Annotated in VFDB_A	341	7.47	341	7.21
Annotated in VFDB_B	535	11.71	547	11.57
Annotated in CARD	203	4.44	204	4.31
Annotated in BacMet_exp	262	5.74	269	5.69
Annotated in BacMet_pre	345	7.55	349	7.38
Annotated in SARG	71	1.55	68	1.44
Annotated in at least one database	4555	99.74	4717	99.75
Total genes	4567	100	4729	100

Overall functional-category distributions were broadly similar between Pa-6 and YSFJ across the general and specialized annotation resources summarized in Figure S3. Detailed structural-annotation statistics, database-specific annotation counts, and functional-category distributions are provided in Table 3, Tables S4–S5, and Supplementary Results SR4.2.

#### Comparative overlap of database annotation IDs between Pa-6 and YSFJ

Pa-6 and YSFJ shared most annotation identifiers across the examined functional and prediction resources, consistent with their broadly similar overall functional profiles (Figure S4(a–g)). Greater strain-associated differences were observed in several specialized annotation sets related to transport and host interaction (Figure S4(h–k); Tables S4–S5).

Because these comparisons were based on nonredundant database annotation identifiers rather than homologous gene clusters or genomic loci, they were treated as complementary functional descriptions and not as direct evidence of gene presence–absence. Detailed database-specific overlap results are provided in Supplementary Results SR4.3. Resistance-associated CARD comparisons are presented separately in Antibiotic inhibition-zone profiles and resistance-associated annotations (Figure S4(l)).

#### Antibiotic inhibition-zone profiles and resistance-associated annotations

The 18 mulberry-derived *P. ananatis* isolates showed heterogeneous inhibition-zone responses across the 36 antibiotics tested, with both antibiotic-dependent and isolate-dependent variation evident in the corrected inhibition-zone profiles (Figure S5(a-b)). Detailed measurements and response classifications are provided in Supplementary Results SR4.4 and the corresponding supplementary dataset.

Resistance-associated genomic annotations were largely shared between Pa-6 and YSFJ. CARD-based comparison identified four Pa-6-associated, 109 shared, and one YSFJ-associated nonredundant annotation IDs (Figure S4(l); Tables S4–S6). Thus, despite limited strain-associated differences, the resistance-associated annotation profiles of the two representative strains were dominated by a large common component.

#### Phylogenomic relationships of mulberry-associated *P. ananatis* strains

Comparative analyses included 41 *P. ananatis* genomes (Table S7). Reciprocal ANI values between Pa-6 and YSFJ ranged from 98.9126% to 98.9369% (Figure 5(a); Supplementary Results SR4.5). Among the three mulberry-associated strains, reciprocal ANI values ranged from 99.0916% to 99.1111% between Pa-6 and LCFJ-001 and from 98.9870% to 99.0003% between YSFJ and LCFJ-001.

**Figure 5.**
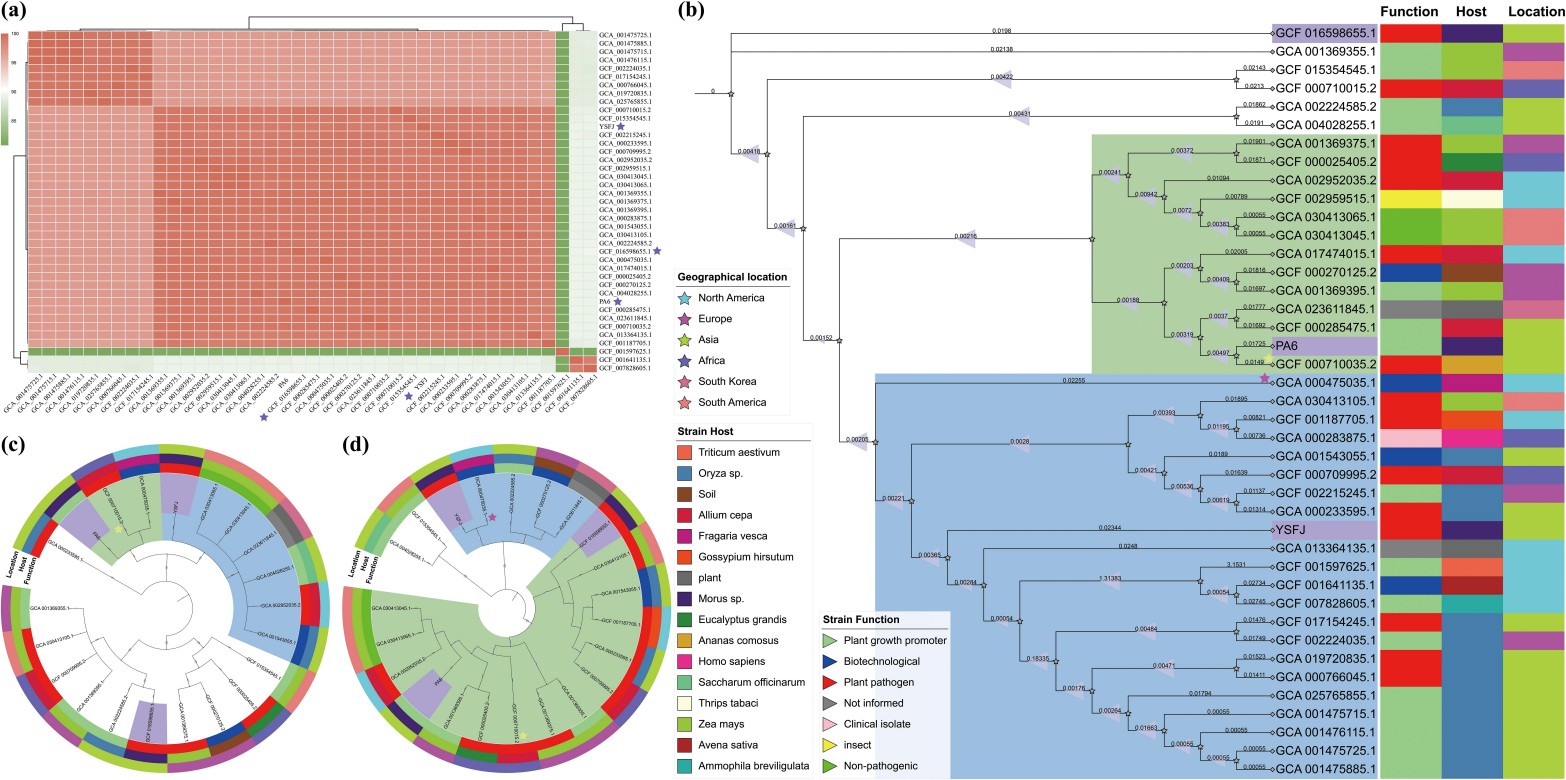
Comparative genomic and phylogenomic relationships among *Pantoea ananatis* genomes.

The comparative genomic dataset comprised 38 publicly available reference genomes, the previously published mulberry-associated strain LCFJ-001, and the two mulberry-associated strains Pa-6 and YSFJ sequenced in the present study. Yellow and pink-purple stars indicate the positions of the reference strains *P. ananatis* LMG 2665 and BRT175, respectively. The colored stars are used solely as visual landmarks to facilitate comparison of strain positions among the trees and do not represent ecological, functional, or phenotypic categories.

(a) Hierarchically clustered heatmap of pairwise average nucleotide identity (ANI) values among all 41 genomes. Rows and columns represent individual genome assemblies. The sequential color scale ranges from 80% to 100% ANI, with higher values indicating greater nucleotide identity.

(b) Phylogenomic tree inferred from concatenated single-copy core-gene sequences from a selected subset of 21 genomes. Green shading highlights the major clade containing Pa-6, whereas blue shading highlights a different major clade containing YSFJ. LCFJ-001 is positioned on a separate branch outside the two highlighted clades. The shaded regions are used only to emphasize the relative positions of the focal strains and do not represent ecological, functional, phenotypic, host-associated, or geographical groups. The accompanying metadata columns indicate the reported ecological or functional category, host or isolation source, and geographical origin of each genome.

(c) Circular phylogenetic tree inferred from 16S rRNA gene sequences from the same 21-genome subset used in panel (b). The surrounding annotation rings indicate the reported ecological or functional category, host or isolation source, and geographical origin of each genome.

(d) Circular phylogenomic tree inferred from whole-genome SNPs across all 41 genomes. The surrounding annotation rings indicate the reported ecological or functional category, host or isolation source, and geographical origin of each genome. Geographical metadata were standardized at the continental level for visualization; South Korean isolates were therefore assigned to Asia. Country-level information, where shown, is retained only as descriptive source metadata. Genome accession numbers and associated metadata are provided in Table S7.

Despite this high nucleotide similarity, Pa-6 and YSFJ were positioned in different major clades in the single-copy core-gene phylogeny (Figure 5(b)). The 16S rRNA gene phylogeny also placed Pa-6, YSFJ, and LCFJ-001 at distinct positions rather than in a single mulberry-associated cluster (Figure 5). In the core-genome SNP phylogeny, Pa-6 and LCFJ-001 occurred within the same broader clade, whereas YSFJ was positioned in a different clade (Figure 5).

Strain-specific gene-content variation was evident across the analyzed genomes (Figure 6(b)). Pa-6 contained 209 strain-specific homologous clusters, whereas YSFJ contained 150. Examination of these clusters together with the corresponding genome annotations identified a limited set of clear gene-level presence–absence differences: a GH19 glycoside-hydrolase locus was detected in Pa-6 but not in YSFJ, whereas wbpZ and feoB were detected in YSFJ but not in Pa-6 (Table S8). Other strain-specific clusters were treated as sequence- or copy-level differences when related sequences or additional copies were present in the opposite genome.

**Figure 6.**
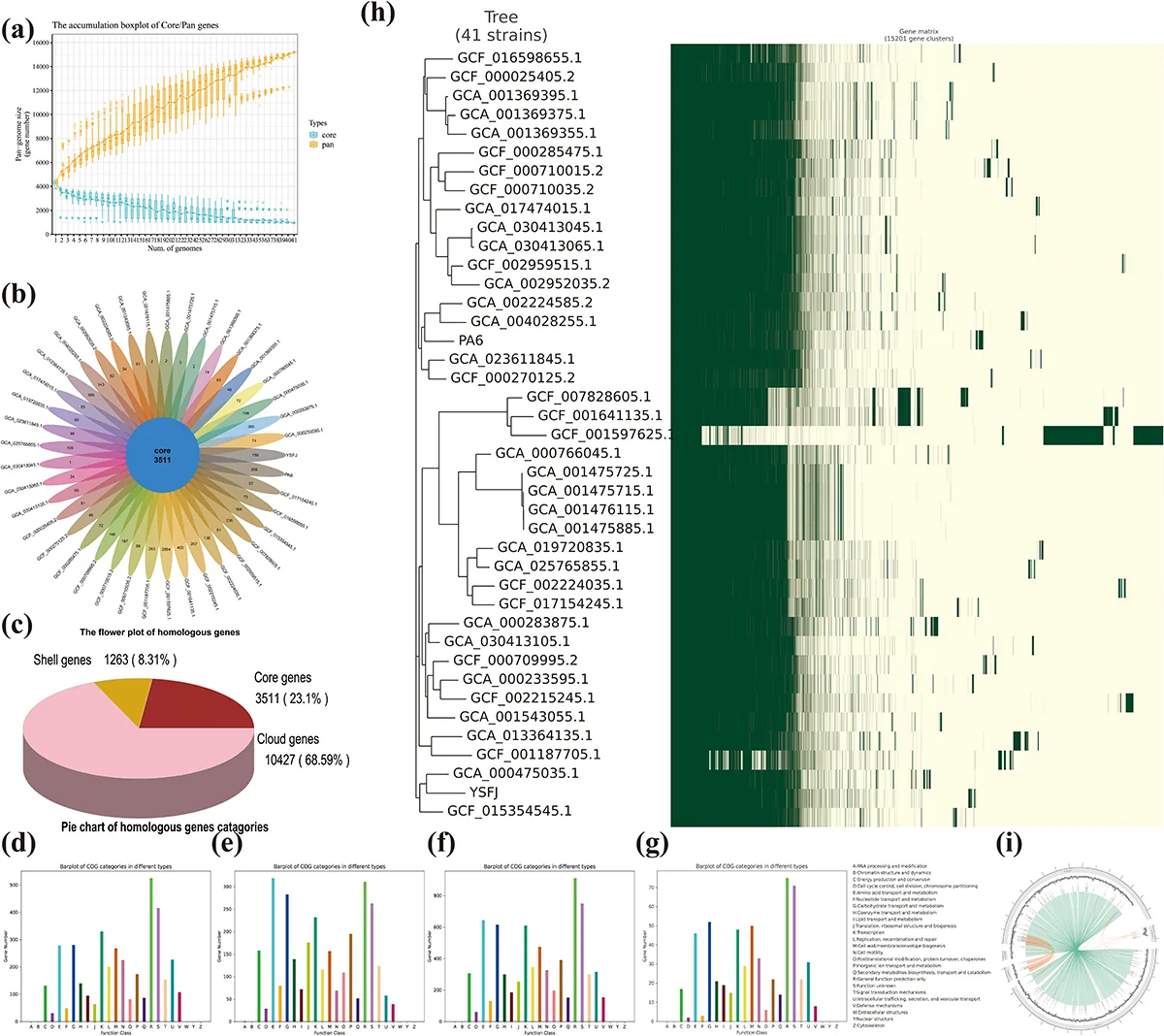
Pan-genome structure and gene-content variation among 41 *Pantoea ananatis* genomes.

The dataset comprised 38 publicly available reference genomes, the previously published mulberry-associated strain LCFJ-001, and the two mulberry-associated strains Pa-6 and YSFJ sequenced in the present study.

(a) Core- and pan-genome rarefaction analysis of the 41 *P. ananatis* genomes. The x-axis represents the number of genomes included in each subset, and the y-axis represents the distribution of homologous gene-cluster counts. Orange and blue indicate pan-genome and core-genome cluster distributions, respectively.

(b) Petal representation of core and strain-specific homologous gene clusters across the 41 *P. ananatis* genomes. The central value represents the 3,511 homologous clusters shared by all analyzed genomes, whereas the value associated with each individual genome represents the number of strain-specific homologous clusters identified for that genome. Pa-6 and YSFJ contained 209 and 150 strain-specific homologous clusters, respectively. The plot is intended as a descriptive overview of gene-content variation; complete cluster assignments and numerical values are provided in Table S8.

(c) Distribution of homologous gene clusters among the core, shell, and cloud categories. The analysis identified 3,511 core, 1,263 shell, and 10,427 cloud gene clusters, resulting in a total pan-genome of 15,201 gene clusters.

(d) COG functional classification of cloud gene clusters. Function unknown and transcription were the largest categories, containing 416 and 330 annotated gene clusters, respectively. The x-axis indicates COG functional categories, and the y-axis indicates the number of annotated gene clusters.

(e) COG functional classification of core gene clusters. Amino acid transport and metabolism, carbohydrate transport and metabolism, and transcription were the largest categories, containing 319, 283, and 232 annotated gene clusters, respectively. The x-axis indicates COG functional categories, and the y-axis indicates the number of annotated gene clusters.

(f) COG functional classification of the complete pan-genome gene-cluster set. General function prediction only and function unknown were the most highly represented categories, containing 911 and 750 annotated gene clusters, respectively. The x-axis indicates COG functional categories, and the y-axis indicates the number of annotated gene clusters.

(g) COG functional classification of shell gene clusters. General function prediction only and function unknown were the largest categories, containing 75 and 71 annotated gene clusters, respectively. The x-axis indicates COG functional categories, and the y-axis indicates the number of annotated gene clusters.

(h) Gene presence–absence matrix for the 41 *P. ananatis*genomes. A separate single-copy core-gene phylogenomic tree reconstructed from all 41 genomes is displayed alongside the matrix and was used to order the genomes. This tree is distinct from the 21-genome single-copy core-gene phylogeny shown in Figure 5(b).

(i) Pairwise whole-genome collinearity analysis between Pa-6 and YSFJ. Links connect corresponding collinear blocks between the two complete genome assemblies. A total of 2,440 alignment blocks were identified, of which 51.51% had nucleotide identities greater than 95%, 42.71% had identities of 80%-95%, and 5.78% had identities below 80%. The percentages indicate the proportions of alignment blocks within the corresponding nucleotide-identity intervals rather than the proportions of the complete genome sequences.

Visual comparison did not reveal an obvious correspondence between the major phylogenomic groupings and the reported ecological or functional categories, host or isolation sources, or geographical origins of the analyzed strains. These metadata were used descriptively and were not equivalent to standardized mulberry phenotypes. Pairwise ANI values for the three focal mulberry-associated strains and detailed phylogenomic relationships are described in Supplementary Results SR4.5.

#### Pan-genome analysis

Pan-genome analysis of the 41 *P. ananatis* genomes identified 15,201 homologous gene clusters, including 3,511 core, 1,263 shell, and 10,427 cloud clusters (Figure 6; Table S8). Rarefaction analysis showed that the pan-genome continued to increase as additional genomes were included, whereas the number of core clusters decreased and tended toward stabilization (Figure 6(a)).

Direct comparison of Pa-6 and YSFJ within the 41-genome Roary matrix identified 3,914 homologous clusters shared by both strains, 556 detected in Pa-6 but not YSFJ, and 661 detected in YSFJ but not Pa-6. Pa-6 and YSFJ were represented by 4,470 and 4,575 clusters, respectively, and 102 shared clusters differed in copy number. These pairwise counts are distinct from the 209 Pa-6-specific and 150 YSFJ-specific clusters detected in only one genome across the complete 41-genome dataset (Table S8).

Cluster-level differences were interpreted with reference to the genome annotations because they may reflect gene-content variation, sequence divergence, clustering boundaries, or copy-number differences. The annotation comparison highlighted a GH19-associated locus in Pa-6 and wbpZ- and feoB-associated loci in YSFJ as putative gene-content differences. These observations were not treated as evidence of causal relationships with the host phenotypes.

Functional classification of the accessory and conserved pan-genome components showed distinct COG-category distributions among the cloud (Figure 6), core (Figure 6(e)), complete pan-genome (Figure 6(f)), and shell gene-cluster sets (Figure 6(g)). Genome-wide presence–absence patterns further illustrated extensive accessory-gene variation across the 41 genomes (Figure 6(h)).

Pairwise whole-genome collinearity analysis showed extensive conserved regions between Pa-6 and YSFJ together with alignment blocks spanning different nucleotide-identity ranges (Figure 6). Detailed COG distributions, presence–absence relationships, and collinearity statistics are provided in Supplementary Results SR4.6.

### Untargeted UPLC-Q-TOF-MS metabolomic profiling of mulberry seedling culture supernatants following *P. ananatis* inoculation

Untargeted UPLC-Q-TOF-MS profiling of culture supernatants collected at 15 dpi showed treatment-associated differences among CK, Pa-6, and YSFJ in both positive- and negative-ionization modes. Both inoculated treatments contained more detectable metabolic features than CK, with the highest feature counts observed in the YSFJ treatment. Corresponding chromatographic profiles are shown in Figure S6(a–f), and detailed feature information is provided in Tables S9–S10 and Supplementary Results SR5.1.

#### Data normalization and global metabolic variation among the CK, Pa-6, and YSFJ treatments

Normalization reduced differences in sample-wise signal distributions in both ionization modes (Figure S7(a–b)). Principal-component analysis showed treatment-associated variation, with distinguishable sample distributions in positive-ionization mode (Figure S7(c)) and separation of CK from the inoculated treatments with partial Pa-6/YSFJ overlap in negative-ionization mode (Figure S7(d)).

Exploratory partial least-squares discriminant analysis also showed treatment-associated separation in both ionization modes (Figure S7(e–f)). Sample-correlation analyses showed high similarity among CK replicates together with treatment-associated and within-treatment variation among the inoculated samples (Figure S7(g–h)), while hierarchical-clustering heatmaps showed distinct but partially overlapping abundance patterns among CK, Pa-6, and YSFJ (Figure S7(i–j)).

Together, these analyses showed that Pa-6 and YSFJ inoculation was associated with changes in the extracellular metabolic profiles of the mulberry seedling culture system. Detailed feature counts and multivariate-analysis results are provided in Supplementary Results SR5.1.

#### Inoculation-associated and treatment-specific metabolic features

Eighteen nonredundant metabolic features detected in both inoculated treatments but excluded from the CK-associated feature set were putatively annotated (Table 4). These annotations represented chemically diverse compounds and were treated as inoculation-associated features rather than as metabolites of confirmed bacterial origin.

**Table 4.** Integrated metabolites identified across three replicates.

Formula	Compound Name	Category
C_5_H_14_NO	Choline	Inoculation-associated
C_9_H_17_NO_5_	Pantothenic Acid	Inoculation-associated
C_10_H_15_NO_4_	N-(3-oxo-hexanoyl)-homoserine lactone	Inoculation-associated
C_10_H_17_NO_4_	2-amino-8-oxo-9,10-epoxy-decanoic acid	Inoculation-associated
C_21_H_44_O	Heneicosanol	Inoculation-associated
C_23_H_46_O	cis-9,10-Epoxytricosane	Inoculation-associated
C_5_H_10_O_4_	2,3-Dihydroxy-3-methylbutyric acid	Inoculation-associated
C_5_H_6_O_4_	1,1-Cyclopropanedicarboxylic acid	Inoculation-associated
C_6_H_10_O_5_	1,5-Anhydro-D-fructose	Inoculation-associated
C_6_H_12_O_4_	2,3-Dihydroxy-3-methylvaleric acid	Inoculation-associated
C_6_H_10_O_4_	3-Hydroxy-3-methyl-2-oxo-pentanoic acid	Inoculation-associated
C_7_H_12_O_5_	3-Isopropylmalate	Inoculation-associated
C_6_H_12_O_3_	Leucinic acid	Inoculation-associated
C_9_H_10_O_4_	cis-3-(3-Carboxyethenyl)-3,5-cyclohexadiene-1,2-diol	Inoculation-associated
C_30_H_32_O_18_	Kaempferol 3-(6 G-malonylneohesperidoside)	Inoculation-associated
C_13_H_14_N_2_O_4_S_2_	Gliotoxin	Inoculation-associated
C_15_H_12_O_6_	2,6,7,4’−Tetrahydroxyisoflavanone	Inoculation-associated
C_39_H_36_O_9_	Chalcomoracin	Inoculation-associated
C_9_H_16_N_2_O_5_	Hydroxyprolyl-Threonine	Pa-6-treatment-specific
C_24_H_30_O_10_	Bidenoside B	Pa-6-treatment-specific
C_5_H_11_NO_2_S_2_	Bis(2-hydroxyethyl)dithiocarbamic acid	Pa-6-treatment-specific
C_10_H_16_O_8_	3-Hydroxy-4-butanolide	Pa-6-treatment-specific
C_14_H_28_O_6_	(S)-3-Octanol glucoside	Pa-6-treatment-specific
C_6_H_11_NO_2_S	trans-S-(1-Propenyl)-L-cysteine	YSFJ-treatment-specific
C_17_H_20_O_10_	5-(3“,5”-Dihydroxyphenyl)-gamma-valerolactone 3-O-glucuronide	YSFJ-treatment-specific
C_5_H_8_N_4_O_3_S_2_	Methazolamide	YSFJ-treatment-specific
C_5_H_7_NO_2_S	3,4-Dehydrothiomorpholine-3-carboxylate	YSFJ-treatment-specific

Note: Features detected in both the Pa-6- and YSFJ-inoculated treatments but not in the uninoculated control (CK) were designated as *Inoculation-associated*. Features detected only in the Pa-6- or YSFJ-inoculated treatment were designated as Pa-6-treatment-specific or YSFJ-treatment-specific, respectively. These designations describe treatment-associated detection patterns and do not establish the biological origin of the corresponding compounds.

In addition, five features were detected only in the Pa-6-inoculated treatment and four only in the YSFJ-inoculated treatment (Table 4). Complete putative annotations and molecular formulas for the inoculation-associated and treatment-specific features are provided in Supplementary Results SR5.2.

#### Relative-abundance differences between the Pa-6- and YSFJ-inoculated treatments

Comparison of the Pa-6- and YSFJ-inoculated treatments identified 335 metabolic features differing in the direction of mean normalized abundance across the positive- and negative-ionization datasets. Of these, 165 showed higher mean abundance in the Pa-6 treatment and 170 showed higher mean abundance in the YSFJ treatment (Figure S8(a–f); Table S11). These directional assignments were independent of statistical significance.

Eighteen features differed between treatments at the nominal significance threshold (*p* < 0.05), including 12 with higher mean abundance in Pa-6 and six with higher mean abundance in YSFJ. After Benjamini–Hochberg correction, three features remained significant at FDR < 0.05. C_24_H_36_O_5_ (FDR = 0.00335) and C_5_H_11_NO_2_S_2_ (FDR = 0.01563) showed higher mean abundance in the Pa-6 treatment, whereas C_5_H_8_N_4_O_3_S_2_ (FDR = 0.00503) showed higher mean abundance in the YSFJ treatment.

Fold-change and volcano-plot analyses visualized the corresponding abundance patterns in both treatment directions and ionization modes (Figure S8(a–f)). Complete feature-level abundance directions, normalized intensities, nominal *p* values, and FDR-adjusted *p* values are provided in Table S11 and Supplementary Results SR5.3.

#### KEGG pathway analysis and pathway-level Co-mapping of Pa-6-associated metabolic features and KOs derived from Pa-6-specific homologous clusters

KEGG pathway analysis of compounds annotated from the 165 metabolic features showing higher mean normalized abundance in the Pa-6 treatment highlighted pathways related to central carbon, amino-acid, organic-acid, and cofactor metabolism, including the citrate cycle, alanine, aspartate and glutamate metabolism, butanoate metabolism, glyoxylate and dicarboxylate metabolism, and nicotinate and nicotinamide metabolism (Figure 7(a)).

**Figure 7.**
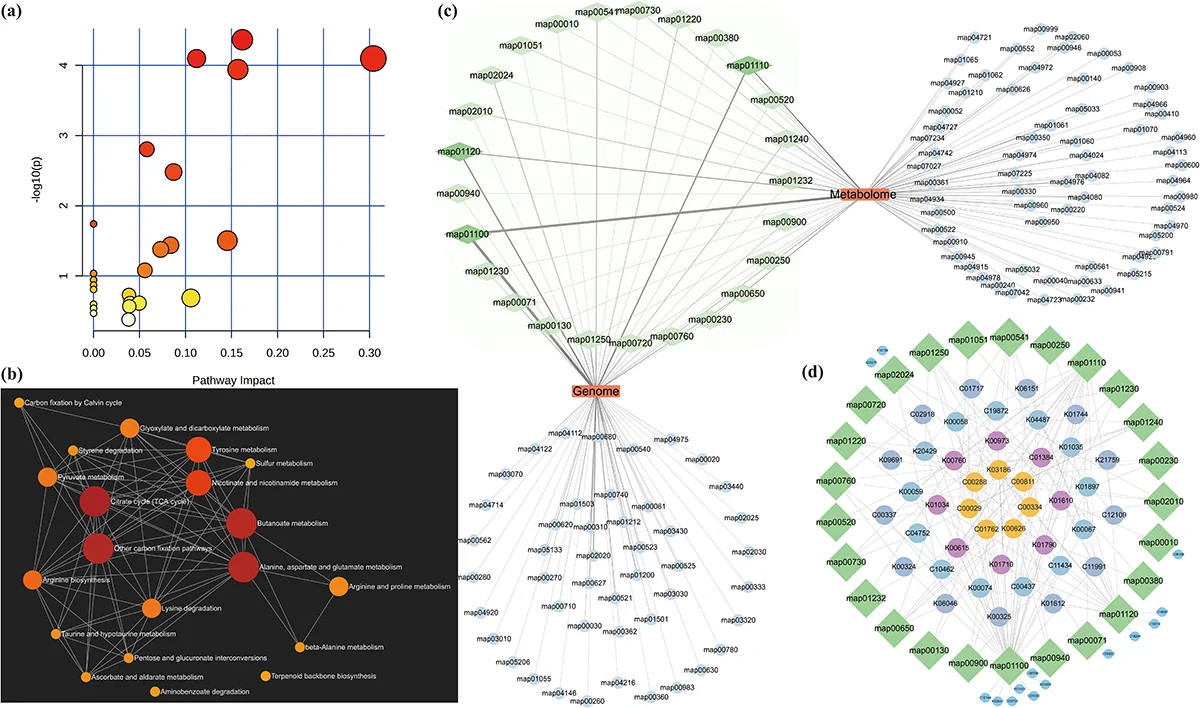
KEGG pathway analysis and pathway-level co-mapping of Pa-6-associated metabolic features and KOs derived from Pa-6-specific homologous clusters.

(a) KEGG pathway analysis based on annotated compounds derived from the 165 metabolite features showing higher mean normalized abundance in culture supernatants from Pa-6-inoculated seedlings than in those from YSFJ-inoculated seedlings. The horizontal axis represents pathway impact, and the vertical axis represents −log_10_ (*p* value). Bubble size indicates pathway impact, whereas bubble color reflects pathway-enrichment significance, with colors shifting from yellow to red as the *p* value decreases.

(b) Metabolite-based KEGG pathway association network. Nodes represent mapped pathways; node size and color correspond to pathway impact and pathway-enrichment significance, respectively. Edges connect pathways sharing mapped KEGG compound entries.

(c) Integrated KEGG pathway-mapping network for the Pa-6-associated metabolomic dataset and KOs derived from Pa-6-specific homologous clusters. Green diamond-shaped nodes represent the 25 KEGG pathways jointly represented by both datasets, whereas peripheral pathway nodes represent pathways mapped by only one dataset. Edge width is proportional to the normalized pathway mapping score, calculated separately for the metabolomic and genomic datasets. For the metabolomic dataset, the score represents the number of valid KEGG compound entries assigned to a pathway relative to the total number of KEGG-mapped compound entries; for the genomic dataset, it represents the number of KOs derived from Pa-6-specific homologous clusters assigned to a pathway relative to the total number of pathway-mapped KOs derived from Pa-6-specific homologous clusters. Values were multiplied by 1,000. Complete pathway scores are provided in Table S12.

(d) Core pathway-level co-mapping network showing KEGG compound entries and KOs derived from Pa-6-specific homologous clusters assigned to the 25 jointly represented pathways. Green diamonds represent KEGG pathways; circular nodes labeled with C numbers represent KEGG compounds, and circular nodes labeled with K numbers represent KOs derived from Pa-6-specific homologous clusters. Edges indicate assignment of a compound or ortholog to the corresponding KEGG pathway. Complete pathway-to-node assignments are provided in Table S13. The networks indicate pathway co-membership and pathway-level convergence between the metabolomic and genomic datasets and do not represent direct gene–metabolite regulatory interactions.

The corresponding metabolite-based pathway association network showed extensive connectivity among central-carbon, amino-acid, organic-acid, and cofactor-related pathways through shared KEGG compound entries (Figure 7(b)).

Pathway-level comparison of Pa-6-associated KEGG compound entries with KOs derived from Pa-6-specific homologous clusters identified 25 jointly represented KEGG pathway categories (Figure 7). These included broad metabolic categories as well as more specific pathways such as glycolysis/gluconeogenesis, alanine, aspartate and glutamate metabolism, butanoate metabolism, nucleotide-sugar metabolism, ABC transporters, and quorum sensing.

The core co-mapping network resolved the compounds and strain-specific-cluster-derived KOs contributing to these 25 shared pathway assignments (Figure 7). Representative KO assignments included *pckA*, *aspA*, *atoA/atoD*, and *pntA/pntB*. These genomic and metabolomic entries occurred within the same KEGG pathway categories and were not interpreted as direct gene–metabolite interactions. Complete pathway scores and pathway-to-node assignments are provided in Tables S12–S13 and Supplementary Results SR5.4.

#### KEGG pathway analysis and pathway-level Co-mapping of YSFJ-Associated metabolic features and KOs derived from YSFJ-Specific homologous clusters

KEGG pathway analysis of compounds annotated from the 170 metabolic features showing higher mean normalized abundance in the YSFJ treatment highlighted pathways involving lipid, amino-acid, cofactor, purine, and aromatic-compound metabolism. Prominent pathways included glycerophospholipid metabolism, pantothenate and CoA biosynthesis, one-carbon pool by folate, glycine, serine and threonine metabolism, β-alanine metabolism, purine metabolism, and aromatic-compound degradation (Figure 8(a)).

**Figure 8.**
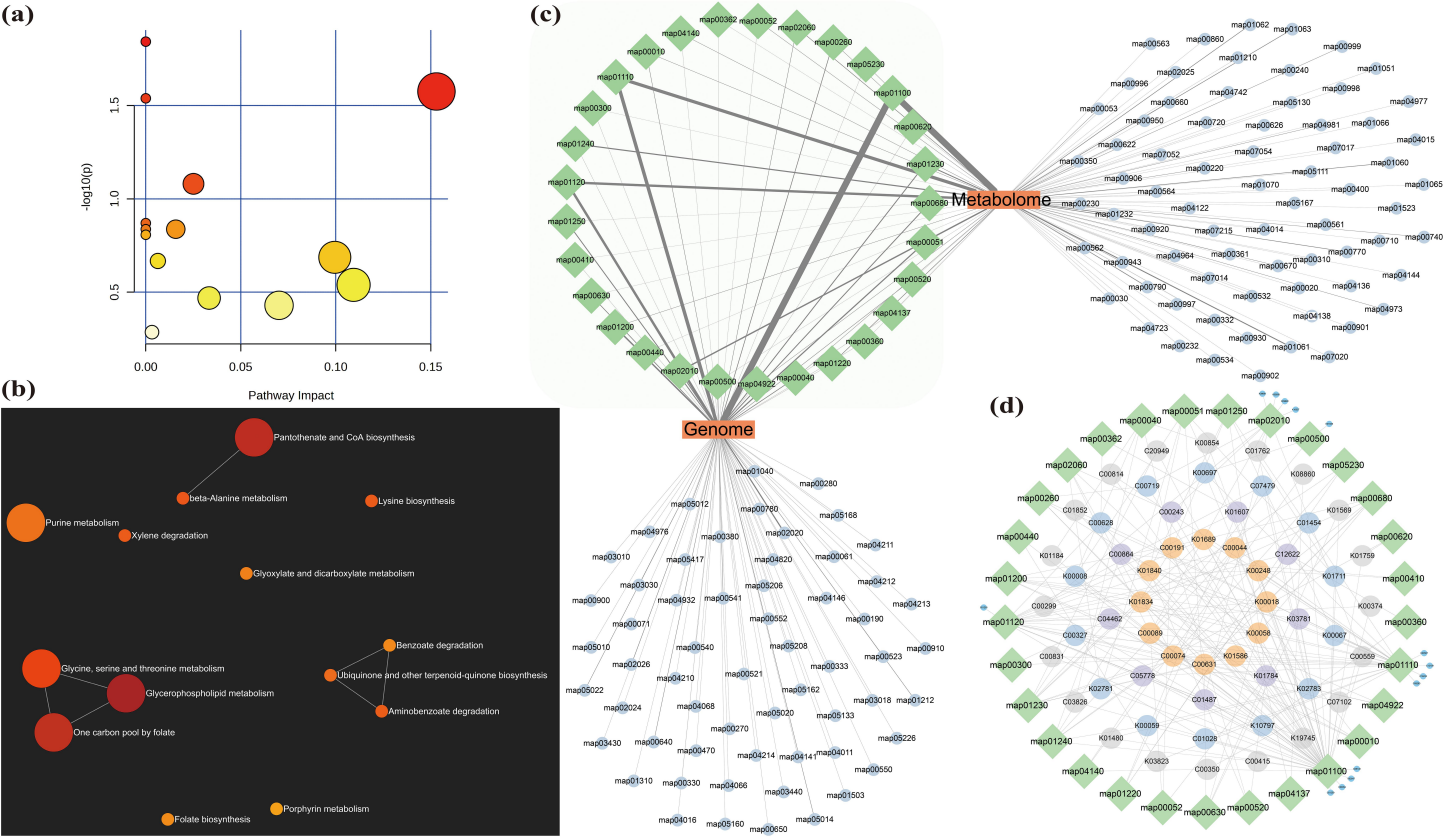
KEGG pathway analysis and pathway-level co-mapping of YSFJ-associated metabolic features and KOs derived from YSFJ-specific homologous clusters.

(a) KEGG pathway analysis based on annotated compounds derived from the 170 metabolite features showing higher mean normalized abundance in culture supernatants from YSFJ-inoculated seedlings than in those from Pa-6-inoculated seedlings. The horizontal axis represents pathway impact, and the vertical axis represents −log_10_ (*p* value). Bubble size indicates pathway impact, whereas bubble color reflects pathway-enrichment significance, with warmer colors corresponding to lower *p* values.

(b) Metabolite-based KEGG pathway association network. Nodes represent mapped KEGG pathways; node size and color correspond to pathway impact and pathway-enrichment significance, respectively. Edges connect pathways sharing mapped KEGG compound entries.

(c) Integrated KEGG pathway-mapping network for the YSFJ-associated metabolomic dataset and YSFJ-treatment-specific genomic dataset. Green diamond-shaped nodes represent the 29 KEGG pathways jointly represented by both datasets, whereas peripheral pathway nodes represent pathways mapped by only one dataset. Edge width is proportional to the normalized pathway mapping score, calculated separately for the metabolomic and genomic datasets. For the metabolomic dataset, the score represents the number of valid KEGG compound entries assigned to a pathway relative to the total number of KEGG-mapped compound entries; for the genomic dataset, it represents the number of KOs derived from YSFJ-specific homologous clusters assigned to a pathway relative to the total number of pathway-mapped KOs derived from YSFJ-specific homologous clusters. Values were multiplied by 1,000. Complete pathway scores are provided in Table S14.

(d) Core pathway-level co-mapping network showing KEGG compound entries and KOs derived from YSFJ-specific homologous clusters assigned to the 29 jointly represented pathways. Green diamonds represent KEGG pathways; circular nodes labeled with C numbers represent KEGG compounds, and circular nodes labeled with K numbers represent KOs derived from YSFJ-specific homologous clusters. Edges indicate assignment of a compound or ortholog to the corresponding KEGG pathway. Complete pathway-to-node assignments are provided in Table S15. The networks indicate pathway co-membership and pathway-level convergence between the metabolomic and genomic datasets and do not represent direct gene–metabolite regulatory interactions.

The corresponding metabolite-based pathway association network showed connectivity among amino-acid, lipid, cofactor, purine, and aromatic-compound metabolism through shared KEGG compound entries (Figure 8(b)).

Pathway-level comparison of YSFJ-associated KEGG compound entries with KOs derived from YSFJ-specific homologous clusters identified 29 jointly represented KEGG pathway categories (Figure 8). These included broad metabolic categories together with more specific pathways involving ABC transporters, the phosphotransferase system, glycolysis/gluconeogenesis, fructose and mannose metabolism, galactose metabolism, glyoxylate and dicarboxylate metabolism, and aromatic-compound degradation.

The jointly represented pathways included functions related to central carbon metabolism, carbohydrate transport and utilization, amino-acid metabolism, membrane transport, cofactor biosynthesis, and aromatic-compound degradation (Figure 8(c,d)). These genomic and metabolomic entries represented KEGG pathway co-membership only. Complete pathway scores and pathway-to-node assignments are provided in Tables S14–S15 and Supplementary Results SR5.5.

## Discussion

### Contrasting Host phenotypes provide a basis for exploring functional divergence

*Pantoea ananatis* encompasses strains with markedly different effects on plants. Pathogenic strains have been reported from onion, rice, strawberry, wheat, tobacco, maize, and other crops [11,92–95], whereas other strains exhibit nutrient-mobilizing, growth-promoting, stress-alleviating, or biocontrol-related traits [5,7,8,96–98]. Contrasting host effects have also been documented among closely related maize-associated strains [4,5,99]. The 18 mulberry-associated isolates examined here extend this pattern of functional heterogeneity to the mulberry system. Their integrated phenotypic profiles resolved two predominant groups, despite all isolates producing indole-3-acetic acid and siderophores and inhibiting *R. pseudosolanacearum* XZqk in vitro (Figure 2). Although similar traits have been reported in plant-beneficial bacteria from other host systems [7–9,96,98], their occurrence in both groups indicates that these individual traits alone cannot explain the contrasting effects on mulberry.

The relationship between phenotypic grouping and MLSA placement further defines this divergence. MLSA provides a useful framework for resolving relationships within *Pantoea* [27], and the two predominant phenotypic groups broadly followed the MLSA topology. However, several isolates did not conform to this overall pattern (Figure 2), indicating that phylogenetic placement was only partially informative about their phenotypes. Similar discrepancies between genetic relatedness and host-associated traits have been reported for onion- and maize-associated *P. ananatis* [4,5,11,12,99]. These findings support examining genomic and metabolic variation alongside phylogenetic relationships when interpreting the contrasting host effects. Pa-6 and YSFJ were selected as representatives of the contrasting phenotypic groups, providing a basis for detailed comparison, although their different phylogenetic backgrounds mean that lineage-associated variation must also be considered.

Pa-6 and YSFJ illustrate this functional contrast most clearly at the whole-plant level. Pa-6-inoculated potted plants remained asymptomatic throughout the 15-d observation period, and the Pa-6 + XZqk treatment showed lower disease severity than the XZqk-alone treatment from 9 dpi onward. In contrast, YSFJ alone caused progressive wilt, despite both strains inhibiting XZqk in vitro (Figure 3). Their contrasting host effects therefore provide information beyond that obtained from plate-based antagonism assays. This is consistent with reports that the relationship between in vitro inhibition and plant protection varies among bacterial biocontrol systems [100–102]. In the present study, shared antagonistic activity was accompanied by markedly different host outcomes, indicating that the capacity to inhibit XZqk in vitro was insufficient to account for the functional distinction between Pa-6 and YSFJ.

The endophytic origin of the isolates provides a biological context for the root-inoculation assays. All 18 isolates were obtained from surface-disinfected mulberry tissues using an endophyte isolation procedure. Four isolates, including YSFJ, originated from roots, whereas Pa-6 originated from stem tissue (Table 1). These records provide direct evidence of root association in the sampled mulberry plants. Roots are an important entry site for many bacterial endophytes, which can enter through natural openings or wounds and subsequently colonize internal plant tissues [103,104]. Endophytic colonization following root inoculation has also been documented experimentally in grapevine inoculated with *Burkholderia* sp. strain PsJN [105]. These studies provide a biological rationale for evaluating mulberry-derived endophytic isolates through root application, including isolates recovered from stems. In the present study, root drenching following standardized root wounding provided a common experimental condition for comparing the host effects of Pa-6 and YSFJ and evaluating protection against XZqk. The isolation records support root association but do not establish recovery from rhizosphere soil, and the inoculation assays do not determine the natural entry routes of the individual strains.

Microscopic observations provided a corresponding tissue-level contrast. YSFJ-inoculated seedlings exhibited bacterial aggregation, xylem occlusion, vascular disorganization, and cellular damage, whereas bacterial cells were more dispersed and no obvious vascular blockage was observed in the examined Pa-6 fields (Figure 4). Previous studies have visualized bacterial cells in *P. ananatis*-associated maize lesions [20] and shown that host injury can facilitate infection in onion [106]. Bacterial accumulation and biofilm development can also impair water transport in *Ralstonia* wilt systems [107–109], providing a physiological context for interpreting the vascular changes observed here. Together, the whole-plant and microscopic observations associate the contrasting Pa-6 and YSFJ phenotypes with different patterns of bacterial distribution and tissue damage. The images are qualitative, however, and do not establish differences in bacterial load or demonstrate that aggregation alone caused wilt.

The expression of these phenotypes also depended on the experimental context. Pa-6 caused comparatively limited damage, but mild yellowing developed at later stages of the axenic-seedling experiment, whereas potted plants remained asymptomatic. Endophytic bacteria can establish neutral or beneficial associations with their hosts, but endophytic occurrence alone does not define the outcome of the interaction [103,104]. Nutrient availability, host physiology, colonization conditions, and environmental stress can modify the effects of plant-associated bacteria [5,8,96,100,110,111]. The beneficial-associated and pathogenic-associated designations used here therefore describe the contrasting responses observed under the tested conditions. This context dependence further supports examining host-contact metabolic patterns alongside genomic differences when interpreting the effects of the two strains.

Taken together, the contrasting host phenotypes, their partial correspondence with MLSA relationships, and the shared occurrence of plant growth-promoting and antagonistic traits make Pa-6 and YSFJ informative representatives for investigating functional divergence in mulberry-associated *P. ananatis*. Their comparison allows us to examine whether accessory-genome composition, strain-associated genomic differences, and host-contact metabolic patterns provide additional insight into the divergent host outcomes beyond that offered by MLSA placement and conventional phenotypic screening. These complementary analyses provide a basis for identifying candidate features associated with functional differentiation, while assessment of their predictive value across the broader isolate collection will require genomic data linked to the measured phenotypes of additional isolates.

### Genomic and metabolic features associated with functional divergence between Pa-6 and YSFJ

#### Phylogenetic background and gene-content differences

Pa-6 and YSFJ showed markedly different effects on mulberry despite their high overall genomic similarity. Their reciprocal ANI values were 98.9126–98.9369%, and both strains had similar overall genome composition. Nevertheless, MLSA and genome-based phylogenies placed them at different positions within *P. ananatis* (Figure 5). A relevant comparative context is provided by studies of onion-associated *P. ananatis*, in which pathogenicity variation and its genomic basis have been extensively investigated. These studies have documented differences in pathogenicity among closely related strains and associated accessory-gene variation with onion-pathogenic phenotypes [11,12]. Maize-associated strains also exhibit growth-promoting, commensal, or pathogenic phenotypes despite close genomic relationships [4,5]. These observations support examining variable genomic features alongside overall relatedness when interpreting the contrasting effects of Pa-6 and YSFJ.

For comparison, Agarwal et al. [12] reported ANI values of 99.0–99.9% among 81 *P. ananatis* strains from onion, weeds, and thrips that were evaluated for onion pathogenicity. The Pa-6–YSFJ values fall slightly below this range, although the strain collections and analytical procedures differ between the studies. Agarwal et al. used CDS-based ANI calculated with GET_HOMOLOGUES, whereas the present study used fastANI on genome assemblies. The numerical difference therefore cannot be attributed to a particular biological or computational factor without a harmonized comparison. The principal relevance of the onion studies is that they demonstrate substantial variation in host-associated phenotypes within a closely related species, providing a framework for considering the contribution of variable gene content in the mulberry-associated strains.

The two strains differed in accessory-gene content as well as genome size. YSFJ had a genome 77,489 bp larger than that of Pa-6 and carried two plasmids, compared with one in Pa-6. Direct comparison within the 41-genome Roary matrix identified 3,914 shared homologous clusters, 556 detected in Pa-6 but not YSFJ, and 661 detected in YSFJ but not Pa-6. These pairwise counts differ in scope from the 209 Pa-6-specific and 150 YSFJ-specific clusters identified as unique to a single genome across the full dataset (Figure 6; Table S8). A cluster absent from one focal strain may still occur in other reference genomes. A large accessory component is characteristic of *P. ananatis* and has been proposed to contribute to its ecological versatility across hosts and environments [2]. Accessory-gene variation has also been associated with pathogenicity differences in phenotyped collections of onion-associated strains [11,12]. The direct Pa-6–YSFJ comparison therefore identifies a set of variable clusters that provides a focused starting point for investigating their different interactions with mulberry, rather than a demonstrated cause of those interactions.

Plasmid content provides another component of this genomic contrast. YSFJ carried an additional 211,717-bp plasmid, whereas Pa-6 contained a single plasmid. Plasmid-borne functions can influence host-associated phenotypes in *P. ananatis*, as elimination of an endogenous plasmid from strain DZ-12 reduced disease severity and altered biofilm formation [13]. Examination of the existing annotations showed that the additional YSFJ plasmid contained sequences associated with chemotaxis, fimbrial or adhesion-related functions, autotransport, and membrane transport (Tables S4–S5). However, T6SS-associated genes were detected on the principal plasmids of both strains, and the highlighted GH19-, *wbpZ*-, and *feoB*-associated loci were not identified among plasmid-encoded CDSs. Plasmid variation thus adds to the differences in accessory-genome composition, while the distribution of these annotations indicates that the contrasting host effects cannot be explained simply by plasmid number.

The public genomes place these differences within the broader diversity of *P. ananatis*. Their reported ecological or functional categories, however, derive from different hosts, assays, and experimental conditions and do not constitute a standardized set of mulberry phenotypes. Their value here is therefore to contextualize genomic diversity rather than to establish gene–phenotype associations in mulberry. Because Pa-6 and YSFJ also occupy different phylogenetic positions, lineage-associated variation cannot be separated from phenotype-associated differences in this two-strain comparison. Only Pa-6 and YSFJ among the 18 phenotypically characterized isolates were newly sequenced for the analyses reported here, with the previously published LCFJ-001 genome included as an additional mulberry-associated reference. Genome-wide comparisons across that collection, particularly of isolates with contrasting phenotypes within the same MLSA group, would provide a stronger basis for assessing these associations. Within its present scope, the direct comparison highlights accessory-gene content and plasmid composition as candidate sources of variation beyond overall nucleotide similarity.

#### Candidate host-interaction and stress-adaptation modules

Pa-6 and YSFJ shared most annotations related to transport, host interaction, and stress response, but YSFJ contained more strain-associated annotation IDs in PHI-base and both VFDB datasets. Pa-6 and YSFJ contained 20 and 22 strain-specific TCDB annotation IDs, 16 and 29 PHI-base IDs, 5 and 12 VFDB Set A IDs, and 65 and 74 VFDB Set B IDs, respectively (Figure S4(h–k)). These values describe nonredundant database identifiers and do not correspond directly to numbers of unique genes or experimentally demonstrated functions. The functional interpretation therefore depends on the individual annotations and their genomic context rather than on the size of the annotation-ID sets alone.

Notable YSFJ-associated differences involved annotations related to cell-surface polysaccharides. The comparison highlighted a *wbpZ*-associated locus in YSFJ and strain-associated differences involving *galE*. WbpZ is a rhamnosyltransferase involved in polysaccharide-antigen biosynthesis in *Pseudomonas aeruginosa* [112], and disruption of *galE* in *Erwinia amylovora* affects extracellular-polysaccharide production, lipopolysaccharide side chains, and virulence [113]. The relevance of this functional system to *P. ananatis* is supported by deletion studies showing that *galU*, *ugd*, *waaC*, *waaG*, and *waaL* contribute to normal LPS formation and virulence in onion [114]. O-antigen structure can also affect attachment, cell–cell aggregation, biofilm development, vascular colonization, and recognition by the host in other plant-associated bacteria [115,116]. These findings make surface-polysaccharide variation a relevant functional context for investigating the bacterial aggregation and vascular changes observed after YSFJ inoculation.

The same comparison should not be read as verified absence of this system from Pa-6. YSFJ-specific homologous clusters contained sequences assigned to *galE*, *manB*, *gmd*, *rfbD*, *wzm*, *wzt*, and *waaL*, several of which belong to families represented in both strains. These annotations are therefore best interpreted as variation within a shared surface-polysaccharide system. Differences in cluster or annotation representation do not establish that every homologous sequence is absent from Pa-6.

A second notable annotation difference involved a *feoB*-associated locus in YSFJ. FeoB participates in ferrous-iron uptake. In *Xanthomonas oryzae* pv. *oryzae*, loss of *feoB* impairs growth under iron limitation and reduces virulence in rice [117]. This functional precedent identifies iron acquisition as another process potentially relevant to bacterial performance in host tissues. The YSFJ-associated annotation therefore provides a candidate for examining differences in iron acquisition during interaction with mulberry, although enhanced uptake or a contribution to the observed host phenotype was not measured here.

Resistance-associated functions were largely shared between Pa-6 and YSFJ, including 109 common CARD annotation IDs and efflux-associated annotations such as *emrA*, *emrB*, *acrB*, and *tolC* (Figure S4(l); Tables S4–S6). Such efflux systems can contribute to tolerance of antibiotics, plant-derived antimicrobial compounds, and other chemical stresses [118–120]. A small number of strain-associated annotations, including *salA*-matched sequences in Pa-6 and *carA*/KpnH-related sequences in YSFJ, were also detected [121,122], but these differences showed no simple correspondence with the inhibition-zone profiles. Phenotypic resistance can depend on expression, membrane permeability, substrate specificity, and regulatory context in addition to gene content [123,124]. The resistance-associated repertoire therefore mainly describes a shared background of predicted chemical-stress responses and does not provide a straightforward explanation for the contrasting host effects.

Among the differences highlighted by these comparisons, the *wbpZ*- and *feoB*-associated loci are of particular interest because their predicted functions are relevant to host interaction. Differences involving *galE* and related annotations extend this interpretation to a broader surface-polysaccharide system. These candidates occur against a substantial background of shared functions, suggesting that the functional contrast may involve variation in particular components of common physiological systems. The priority of these candidates, and of the Pa-6-associated GH19 locus, is taken up in Interpretation of Candidate genomic differences.

#### Genomic and Metabolomic pathway patterns associated with contrasting functional phenotypes

The extracellular metabolite profiles differed between the Pa-6- and YSFJ-inoculated treatments after 15 d of interaction with mulberry seedlings. Of the 335 features retained for comparison, 165 had higher mean normalized abundance in the Pa-6 treatment and 170 in the YSFJ treatment; 18 reached the nominal significance threshold, and three remained significant after FDR correction (Figure S8; Table S11). When these direction-defined feature sets were mapped to KEGG together with ortholog assignments from strain-specific homologous clusters, 25 pathway categories were jointly represented for Pa-6 and 29 for YSFJ (Figure 7 and 8; Tables S12–S15). These counts describe shared pathway assignments rather than numbers of significantly altered pathways. The purpose of this exploratory comparison was to identify physiological processes represented in both datasets and place the genomic differences within the chemical context of host contact.

This interpretation is consistent with what is known about the chemical environment surrounding plant roots and tissues. Sugars, organic acids, and amino acids released by plants can influence bacterial nutrient use, chemotaxis, biofilm formation, and establishment. Root-derived sugars support rhizosphere colonization by *Pseudomonas*, while malic acid and other organic acids can promote recruitment, chemotaxis, biofilm formation, and root colonization by *Bacillus* [125–127]. Exometabolomic studies have further shown that bacterial substrate preferences vary with root-exudate chemistry, and root-derived amino acids can promote bacterial germination and colonization [128,129]. The host-contact medium used here therefore samples a chemical environment relevant to bacterial interaction with living mulberry, although its composition can reflect both plant and bacterial processes.

In the Pa-6 treatment, the mapped pathways included central-carbon metabolism, alanine, aspartate and glutamate metabolism, butanoate metabolism, glyoxylate and dicarboxylate metabolism, purine metabolism, and nicotinate and nicotinamide metabolism (Figure 7(a,b)). Pa-6-specific homologous clusters contained sequences assigned to *pckA*, *aspA*, *paaH*, *atoA*, *atoD*, *hprT*, *pntA*, and *pntB*, and several of these assignments fell within the same pathways as Pa-6-treatment-associated metabolic features (Figure 7(c,d); Tables S12–S13). This combination draws attention to organic-acid utilization, amino-acid metabolism, acetyl-CoA-linked reactions, and redox balance as physiological contexts for interpreting the Pa-6-associated patterns.

The redox component is of particular interest. *pntA* and *pntB* encode the membrane-bound transhydrogenase PntAB, which contributes substantially to cellular NADPH generation and redox-cofactor balance in *Escherichia coli* [130]. Because these gene families occur in both Pa-6 and YSFJ, their assignment to strain-specific clusters does not indicate that either strain lacks the system. Nevertheless, their representation alongside *pckA*, *aspA*, *paaH*, and *atoA*/*atoD* places carbon metabolism and redox homeostasis among the processes relevant to the comparison. This interpretation is consistent with the broad metabolic versatility reported for the *P. ananatis* accessory genome [2]. In the present study, these patterns occurred alongside comparatively limited tissue damage and the protective effect observed in the Pa-6 + XZqk treatment, providing a basis for considering nutrient utilization and metabolic adjustment as candidate components of the Pa-6 phenotype.

YSFJ showed a different pattern of pathway representation. Its treatment-associated features mapped to glycolysis/gluconeogenesis, fructose and mannose metabolism, amino-sugar and nucleotide-sugar metabolism, PTS-mediated carbohydrate uptake, ABC transporters, and aromatic-compound metabolism (Figure 8(a,b)). YSFJ-specific homologous clusters included sequences assigned to *galE*, *manB*, *xylB*, *katE*, and *otsA*, together with PTS- and ABC-transporter-related orthologs (Figure 8(c,d); Tables S14–S15). These assignments highlight carbohydrate uptake and utilization, membrane transport, surface-polysaccharide precursor metabolism, and stress responses within the YSFJ-associated dataset. They describe the functional categories represented in the comparison, rather than functions necessarily absent from Pa-6.

The representation of carbohydrate transport may be relevant to bacterial performance in host tissues. The phosphotransferase system links carbohydrate uptake and phosphorylation with broader control of carbon metabolism [131], whereas ABC transporters mediate acquisition of sugars, amino acids, and mineral nutrients in plant-associated bacteria [132]. Plant carbohydrates can also affect bacterial physiology beyond nutrition; glucose and galactose, for example, influence the production of ralfuranone/ralstonin metabolites in *Ralstonia solanacearum* [133]. The representation of PTS, ABC transport, and carbohydrate-metabolism functions in the YSFJ-associated data therefore identifies substrate acquisition and utilization as relevant processes for comparing the two strains under host-contact conditions.

The YSFJ-associated data also pointed to functions relevant to environmental-stress responses. In *R. solanacearum*, hydroxycinnamic-acid degradation increases tolerance to antimicrobial plant phenolics and contributes to root colonization and virulence, whereas salicylic-acid degradation reduces chemical toxicity during infection [134,135]. Trehalose synthesized through OtsA contributes to osmotic-stress tolerance and fitness during plant infection [136]. The YSFJ-specific homologous-cluster set also contained a sequence assigned to *katE*. In *R. solanacearum*, KatE contributes to hydrogen-peroxide tolerance, although disruption of *katE* did not markedly reduce virulence under the conditions tested [137]. These functional precedents place chemical, osmotic, and oxidative stress responses among the processes relevant to the YSFJ-associated annotations, without establishing equivalent activities or roles in mulberry.

The joint pathway assignments provide exploratory context for the contrasting treatments but do not establish differential pathway activity. The feature sets were defined by the direction of mean normalized abundance rather than statistical significance, and only three of the 335 compared features remained significant after FDR correction. Moreover, extracellular samples were collected at 15 dpi, when host damage differed between treatments. The profiles may therefore reflect host physiological responses, tissue leakage, bacterial metabolism, and their combined effects. Excluding features consistently detected in CK does not establish a bacterial origin for the retained compounds, and the *E. coli* pathway library provides a mapping framework rather than evidence of pathway activity in *P. ananatis*. The resulting patterns offer complementary physiological context for the genomic differences, while pathway co-membership alone does not demonstrate metabolic flux or direct gene–metabolite regulation.

#### Interpretation of Candidate genomic differences

The comparative analyses highlight a GH19-associated locus in Pa-6 and *wbpZ*- and *feoB*-associated loci in YSFJ as the primary candidates for further investigation. These loci were identified as putative gene-content differences in the annotation comparison, whereas several other strain-associated clusters contained annotations from gene families represented in both genomes. They are prioritized because their predicted functions can be linked to the host assays, not because absence of the corresponding genes has been independently confirmed. Cluster and annotation evidence should not be equated with experimentally demonstrated functional differences.

For YSFJ, the *wbpZ*-associated locus is the leading candidate in this set because it occurs alongside the wider group of surface-polysaccharide annotations discussed in Candidate host-interaction and stress-adaptation modules, including sequences assigned to *galE*, *manB*, *gmd*, *rfbD*, *wzm*, *wzt*, and *waaL*. The functional evidence for LPS-associated genes in *P. ananatis* [114], the role of WbpZ in rhamnose-containing polysaccharide biosynthesis [112], and the influence of O-antigen composition on host-associated traits in other bacteria [115] collectively support considering these annotations as part of one cell-surface system. This interpretation links the genomic comparison to the aggregation and vascular changes observed in mulberry, while the contribution of any individual locus remains unresolved.

The *feoB*-associated difference is the second YSFJ-associated priority, through ferrous-iron acquisition. Its documented contribution to growth under iron limitation and virulence in *X. oryzae* pv. *oryzae* supports the biological relevance of this process during host interaction [117], although uptake and any contribution to the mulberry phenotype were not measured here. Other YSFJ-associated annotations are better treated as secondary candidates. Sequences assigned to *otsA* and *katE* extend the comparison to osmotic and oxidative stress responses [136,137]. Strain-associated sequences assigned to polygalacturonase and *pcaC* likewise extend it to plant cell-wall components and aromatic-compound metabolism. *P. ananatis* genomes encode putative enzymes associated with modification or degradation of plant cell-wall components, including polygalacturonases, cellulases, xylanases, and other pectin-associated functions [3], and hydroxycinnamic-acid degradation in *R. solanacearum* promotes tolerance to antimicrobial plant phenolics and contributes to host colonization and virulence [134]. These activities and substrates were not determined in the present study, and the annotations do not establish a hierarchy of causal contributions to the YSFJ phenotype.

For Pa-6, the GH19-associated locus is the leading candidate relevant to microbial interactions. GH19 proteins include both chitinases and endolysin/peptidoglycan-hydrolase proteins, so family membership alone does not resolve substrate specificity or ecological function [138]. Its identification in the Pa-6-associated annotation set warrants further functional consideration, but it does not establish a mechanism for antagonism against XZqk. Both Pa-6 and YSFJ inhibited XZqk in vitro, indicating that the different host outcomes cannot be attributed simply to the occurrence of this annotation.

A broader group of Pa-6-associated annotations, linked to nutrient mobilization and carbon metabolism, is treated as secondary. Pa-6-specific homologous clusters contained sequences assigned to *appA*, gluconate 2-dehydrogenase, *pckA*, *aspA*, *paaH*, *atoA*, and *atoD*. Comparable nutrient-mobilization functions have been discussed in *Pantoea phytostimulans* C3 [139], while phosphate-solubilizing *Pantoea brenneri* isolates produce organic acids during solubilization of insoluble phosphate sources [140]. Nutrient competition provides another relevant functional precedent: a non-disease-inducing *P. ananatis* strain suppressed rice bacterial leaf blight, with co-culture results supporting a role for nutrient competition [141]. These studies support considering nutrient use as a candidate component of bacterial competition under host-associated conditions. In Pa-6, the corresponding annotations and organic-carbon pathway patterns provide a basis for this interpretation, but do not establish nutrient competition as the mechanism underlying protection against XZqk.

Redox balance is a further secondary connection between the Pa-6-associated genomic and metabolic observations. Sequences assigned to Pa-6-specific clusters included *pntA* and *pntB*, whose encoded transhydrogenase contributes to NADPH generation and redox-cofactor balance in *E. coli* [130]. As these families occur in both focal strains, the distinction concerns their representation in different clusters rather than simple presence or absence of PntAB. Their association with central-carbon and organic-acid pathway assignments makes redox homeostasis a relevant aspect of the comparative interpretation, although differences in expression or physiological activity remain to be established.

The resulting priority is therefore limited. The GH19-associated locus in Pa-6 and the *wbpZ*- and *feoB*-associated loci in YSFJ are the first annotations to evaluate, because they were the clearest putative gene-content differences and can be linked to the contrasting phenotypes observed here. Surface-polysaccharide variation, *otsA*- and *katE*-associated stress responses, and plant-cell-wall and aromatic-compound annotations in YSFJ, together with nutrient-mobilization and *pntA*/*pntB*-associated redox functions in Pa-6, form a second group. Targeted disruption and complementation would provide a direct test of these candidates, but such experiments lie beyond the scope of the present study. The sampling design also limits interpretation: the two focal strains differ in phylogenetic background, the public genomes lack a standardized set of mulberry phenotypes, and the comparison cannot separate all lineage-associated variation from variation associated with host responses. Within its current scope, the study identifies biologically relevant genomic differences and complementary host-contact metabolic patterns that help frame functional divergence, while the causal contributions and broader predictive value of individual candidates remain to be established.

## Conclusion

Mulberry-associated *Pantoea ananatis* isolates exhibited substantial variation in host-associated phenotypes. Pa-6 caused comparatively limited tissue damage and was associated with lower disease severity than XZqk alone during the later stages of co-inoculation, whereas YSFJ caused progressive wilt accompanied by bacterial aggregation, xylem occlusion, vascular disorganization, and tissue damage. These outcomes were observed under the specified inoculation conditions and observation periods.

The two focal strains differed in gene-cluster representation, plasmid composition, and host-contact extracellular metabolic profiles despite high overall nucleotide similarity. Annotation-based differences, including those involving GH19-, wbpZ-, and feoB-associated loci, and exploratory pathway assignments provide hypotheses for further investigation. Their relationships to host phenotype remain to be resolved because the comparison involves different phylogenetic backgrounds and does not establish genotype–phenotype associations across the full mulberry isolate collection.

## Supplementary Material

Supplementary_Information-clean.docx

Supplementary Figure.docx

Supplementary Table.xlsx

## Data Availability

The data supporting this study are available in the 4TU.ResearchData repository under DOI: https://data.4tu.nl/datasets/51da0e25-3c0f-48b8-93b0-1b0b0c3f0cdb (Version 4 [142];). The deposited materials include raw and processed datasets, genome assemblies and annotations, comparative-genomic and metabolomic outputs, phenotypic and inoculation data, source data for figures and supplementary materials, and available computational-analysis records. Custom scripts from the original analyses were not retained; available software versions, recorded parameters, input tables, intermediate files, and outputs are provided to support verification of the reported analyses.

## Open Scholarship

This article has earned the Center for Open Science badges for Open Data and Open Materials through Open Practices Disclosure. The data and materials are openly accessible at https://data.4tu.nl/datasets/51da0e25-3c0f-48b8-93b0-1b0b0c3f0cdb
