# Supplementary material for: Functional divergence within mulberry-associated Pantoea ananatis: Insights from comparative genomics and host-contact metabolomics
Source: Virulence. 2026 Nov 7;17(1):2749997. doi: 10.1080/21505594.2026.2749997 (PMC13757542; doi:10.1080/21505594.2026.2749997)
Supplement: Supplementary file 1 [file KVIR_A_2749997_SM3092.docx]

**Supplementary Figures**

**Figure S1. Scanning electron microscopy of 18 mulberry-associated *Pantoea ananatis* isolates**

Scanning electron microscopy images showing the cellular morphology of the 18 isolates. Isolates 1–18 correspond to Pa-1, Pa-2, Pa-3, Pa-4, Pa-5, Pa-6, Pa-7, Pa-8, Pa-9, Pa-10, Pa-11, Pa-12, LCFJ-001, YSFJ, YZFJ, JSFJ, YDFJ2, and YDFJ6, respectively. All isolates exhibited rod-shaped cells, with cell lengths of approximately 500–1,800 nm and widths of 300–600 nm under the examined conditions.

**Figure S15 Figure S2. Sequencing quality, genome-quality assessment, GC–depth profiles, and circular genome maps of Pa-6 and YSFJ.**
Pa-6 and YSFJ are shown on the left and right, respectively, in panels containing paired plots.
**(a)** Per-base Phred quality scores for A, T, C, and G across the paired-end short reads. Positions 1–150 correspond to read 1, and positions 151–300 correspond to read 2.
**(b)** CheckM assessment of the Pa-6 and YSFJ genome assemblies, showing the distributions of single-copy marker genes, missing markers, strain heterogeneity, and estimated contamination.
**(c)** BUSCO assessment of genome completeness. Complete BUSCOs accounted for 100% of the evaluated orthologs in both assemblies. Pa-6 contained 98.39% single-copy and 1.61% duplicated BUSCOs, whereas YSFJ contained 99.19% single-copy and 0.81% duplicated BUSCOs. No fragmented or missing BUSCOs were detected.
**(d)** GC–depth distributions of the Pa-6 and YSFJ assemblies. The central scatter plots show sequencing depth as a function of GC content, while the upper and lateral histograms show the corresponding GC-content and depth distributions. Red dashed lines indicate the mean GC content and mean sequencing depth.
**(e)** Circular genome maps of Pa-6 and YSFJ. Pa-6 comprises one chromosome and one plasmid, whereas YSFJ comprises one chromosome and two plasmids. The circular tracks show genomic coordinates and the distributions of annotated genomic features, COG functional assignments, GC content, and GC skew, as indicated in the accompanying legends.

**Figure S3. Functional annotation profiles of the Pa-6 and YSFJ genomes.**
**(a)** Proportions of coding sequences (CDSs) annotated against the NR, COG, CDD, PFAM, GO, KEGG, CAZy, TCDB, PHI, VFDB_A, VFDB_B, CARD, BactMet_exp, BactMet_pre, and SARG databases. Database names are shown on the x-axis, and the percentage of annotated CDSs is shown on the y-axis. Pa-6 and YSFJ are presented separately.
**(b)** COG functional classification of the annotated CDSs in Pa-6 and YSFJ. COG functional categories are shown on the x-axis, and the number of CDSs assigned to each category is shown on the y-axis.
**(c)** GO classification of the annotated CDSs according to the three principal ontologies: biological process, cellular component, and molecular function. GO terms are shown on the x-axis, with the percentage of annotated CDSs indicated on the left y-axis and the corresponding gene number indicated on the right y-axis.
**(d)** KEGG functional classification of the annotated CDSs in Pa-6 and YSFJ. KEGG pathway categories are shown on the x-axis, and the number of assigned CDSs is shown on the y-axis.
**(e)** NR-based species distribution of homologous sequence matches for Pa-6 and YSFJ. Sector size represents the proportion of annotated CDSs showing homology to each species. *Pantoea ananatis* was the predominant matched species, accounting for 3,434 annotated genes in Pa-6 and 3,477 annotated genes in YSFJ.

**Figure S4. Comparative overlap of database-specific annotation identifiers between the Pa-6 and YSFJ genomes.**
Venn diagrams show database-specific annotation identifiers (IDs) detected in Pa-6 and YSFJ across 18 annotation and prediction resources. In each panel, the left non-overlapping sector represents IDs detected only in Pa-6, the overlapping sector represents IDs shared by Pa-6 and YSFJ, and the right non-overlapping sector represents IDs detected only in YSFJ. The numbers represent unique database annotation IDs rather than numbers of annotated CDSs. The accompanying bar plots summarize the numbers of IDs in each strain, and the lower bars indicate whether IDs were detected in one or both genomes.
**(a)** NR, non-redundant protein sequence database.
**(b)** COG, Clusters of Orthologous Groups.
**(c)** CDD, Conserved Domain Database.
**(d)** PFAM, Protein Families database.
**(e)** GO, Gene Ontology.
**(f)** KEGG, Kyoto Encyclopedia of Genes and Genomes.
**(g)** CAZy, Carbohydrate-Active enZYmes database.
**(h)** TCDB, Transporter Classification Database.
**(i)** PHI, Pathogen–Host Interactions database.
**(j)** VFDB_A, core virulence-factor dataset.
**(k)** VFDB_B, full virulence-factor dataset.
**(l)** CARD, Comprehensive Antibiotic Resistance Database.
**(m)** BactMet_exp, experimentally confirmed biocide- and metal-resistance annotations.
**(n)** BactMet_pre, predicted biocide- and metal-resistance annotations.
**(o)** SARG, Structured Antibiotic Resistance Gene database.
**(p)** GI, predicted genomic-island annotation IDs.
**(q)** PHASTEST, predicted prophage annotation IDs.
**(r)** AntiSMASH-predicted biosynthetic gene cluster IDs. Six cluster IDs were shared by Pa-6 and YSFJ and were assigned to hserlactone, redox cofactor, thiopeptide, siderophore, arylpolyene, and terpene cluster types.

**Figure S5. Antibiotic inhibition-zone profiles of 18 mulberry-derived *Pantoea ananatis* isolates.**
Inhibition-zone diameters were determined from three independent biological replicates for each isolate–antibiotic combination. The mean inhibition-zone diameter was classified using the study-defined thresholds as low inhibition (≤10 mm), intermediate inhibition (11–19 mm), or high inhibition (≥20 mm).
**(a)** Ridgeline plots showing the distributions of inhibition-zone diameters for the 18 isolates against 35 antibiotics. Antibiotics are grouped according to antibiotic class, and the x-axis indicates inhibition-zone diameter in millimeters.
**(b)** Heatmap showing the inhibition category assigned to each isolate–antibiotic combination based on the mean of the three independent biological replicates. Green, yellow, and orange indicate low, intermediate, and high inhibition, respectively. The isolates included Pa-1–Pa-12, LCFJ-001, YSFJ, YZFJ, JSFJ, YDFJ2, and YDFJ6.

**Figure S6. Total-ion chromatograms of mulberry seedling culture supernatants in positive- and negative-ionization modes.**
Supernatants were collected after 15 d from uninoculated mulberry seedling cultures (CK) and cultures inoculated with *Pantoea ananatis* Pa-6 or YSFJ. (a) CK, positive-ionization mode; (b) CK, negative-ionization mode; (c) Pa-6-inoculated treatment, positive-ionization mode; (d) Pa-6-inoculated treatment, negative-ionization mode; (e) YSFJ-inoculated treatment, positive-ionization mode; and (f) YSFJ-inoculated treatment, negative-ionization mode.

**Figure S7. Data-normalization diagnostics and global metabolic variation among the CK, Pa-6, and YSFJ treatments.**
Each treatment comprised three biological replicates. (a, b) Density distributions and sample-wise boxplots of metabolic-feature intensities before and after normalization in positive-ionization (a) and negative-ionization (b) modes. (c, d) Principal-component analysis score plots for the positive-ionization (c) and negative-ionization (d) datasets. The percentages shown on the axes indicate the variance explained by the corresponding principal components. (e, f) Partial least-squares discriminant analysis score plots for the positive-ionization (e) and negative-ionization (f) datasets. (g, h) Sample-correlation heatmaps for the positive-ionization (g) and negative-ionization (h) datasets. (i, j) Hierarchical-clustering heatmaps of metabolic features detected in positive-ionization (i) and negative-ionization (j) modes. Rows represent metabolic features, and columns represent the CK, Pa-6, and YSFJ treatments. Red and blue indicate relatively higher and lower normalized feature intensities, respectively.

**Figure S8. Relative-abundance comparison of metabolic features between the Pa-6- and YSFJ-inoculated treatments.**

Fold-change direction was expressed as Pa-6 relative to YSFJ. Positive values and Up classifications indicate higher mean normalized abundance in the Pa-6 treatment, whereas negative values and Down classifications indicate higher mean normalized abundance in the YSFJ treatment. (a, b) Fold-change distributions of features detected in positive-ionization (a) and negative-ionization (b) modes. (c, d) Univariate statistical comparisons in positive-ionization (c) and negative-ionization (d) modes. (e, f) Volcano plots showing the direction, magnitude, and nominal statistical significance of the differences in positive-ionization (e) and negative-ionization (f) modes. Each treatment comprised three biological replicates. Complete results are provided in Table S9, with feature-level statistical comparisons and FDR-adjusted P values summarized in Table S11.
