# Supplementary material for: Functional divergence within mulberry-associated Pantoea ananatis: Insights from comparative genomics and host-contact metabolomics
Source: Virulence. 2026 Nov 7;17(1):2749997. doi: 10.1080/21505594.2026.2749997 (PMC13757542; doi:10.1080/21505594.2026.2749997)
Supplement: Supplementary file 2 [file KVIR_A_2749997_SM3093.docx]

**Supplementary Information**

# **Supplementary Methods**

## **Supplementary Methods S1. Bacterial isolation, plant preparation, and nucleic acid extraction**

### **S1.1 Bacterial isolation and purification**

Mulberry tissues were surface-sterilized by immersion in 70% ethanol for 30 s and 2% sodium hypochlorite for 2 min, followed by three rinses with sterile distilled water. The sterilized tissues were homogenized in phosphate-buffered saline (PBS), serially diluted, and spread onto Luria-Bertani (LB) agar plates. Plates were incubated at 28°C for 48 h. Colonies with distinct morphologies were selected and repeatedly streaked on LB agar until pure cultures were obtained.

Purified isolates were preliminarily identified by amplification and sequencing of the 16S rRNA gene using the primer pair listed in Table 2. Isolates provisionally assigned to *P. ananatis* were retained for subsequent multilocus sequence analysis and phenotypic characterization. All isolates were stored at −80°C in medium supplemented with 20% (v/v) glycerol.

### **S1.2 Seed sterilization, germination, and axenic-status screening**

Seeds of the mulberry cultivar ‘Gui Sang 12’ (*Morus atropurpurea*) were immersed in 70% ethanol for 3 min and subsequently treated with 0.1% HgCl₂ for 15 min. Seeds were rinsed five times with sterile distilled water and germinated on MS medium at 25°C under a 12-h light/12-h dark photoperiod.

Seedlings used for axenic inoculation and microscopic observation experiments were maintained on sterile MS medium for 60 d. Before inoculation, randomly selected seedlings from each batch were aseptically homogenized, and the resulting tissue suspensions were plated on LB agar and incubated at 28°C for 48 h. Only batches showing no colony growth were used. Uninoculated seedlings maintained under the same aseptic conditions served as negative controls.

### **S1.3 Preparation of seedlings for whole-plant assays**

Seedlings used in the potted-plant assays were transferred to soil autoclaved at 121°C for 15 min. The transplanted seedlings were acclimated under a 16-h light/8-h dark photoperiod and 70% relative humidity before inoculation.

### **S1.4 Preparation of mulberry branch explants**

Branch explants were collected from 15-year-old mulberry trees of the cultivar ‘Lun Jiao 40’. Explants were surface-sterilized in 0.5% sodium hypochlorite for 15 min, rinsed three times with sterile distilled water, and transferred to liquid MS medium. Explants were maintained at 25°C and 85% relative humidity, with the medium renewed daily.

### **S1.5 Bacterial culture and genomic DNA extraction**

For genomic DNA extraction, bacterial strains were cultured in nutrient broth medium (Guangdong Huankai Microbial Science and Technology Co., Ltd., Guangzhou, China) at 28°C for 48 h. Bacterial cells were collected, and genomic DNA was extracted using the Ezup Column Bacteria Genomic DNA Purification Kit (Sangon Biotech, Shanghai, China) according to the manufacturer’s instructions.

## **Supplementary Methods S2. PCR amplification, Sanger sequencing, and sequence processing for MLSA**

### **S2.1 PCR amplification**

Partial sequences of the 16S and 23S rRNA genes, *gyrB*, *leuS*, *pleD*, and *rpoB* were amplified from genomic DNA of all 18 *P. ananatis* isolates. Primer sequences, expected product sizes, locus-specific annealing temperatures, and corresponding sources are provided in Table 2.

PCR amplification was performed in 25-μL reaction mixtures containing 2× Taq Master Mix, forward and reverse primer solutions at 10 μM, 10–50 ng genomic DNA, and nuclease-free water. The thermal cycling program consisted of initial denaturation at 94°C for 3 min; 30 cycles of denaturation at 94°C for 30 s, locus-specific annealing at 55 ± 5°C for 30 s, and extension at 72°C for approximately 1 min kb⁻¹; followed by a final extension at 72°C for 5 min.

### **S2.2 Sanger sequencing and sequence processing**

PCR products were sequenced bidirectionally by Sanger sequencing (Sangon Biotech, Shanghai, China). Forward and reverse reads were assembled and manually edited using BioEdit. Each locus was aligned separately using ClustalW and trimmed to a common alignment length. The resulting sequences were concatenated in the same order for all isolates: 16S rRNA–23S rRNA–*gyrB*–*leuS*–*pleD*–*rpoB*.

### **S2.3 Reference-strain selection**

Nine publicly available *P. ananatis* reference strains were retrieved from the NCBI nucleotide database and included together with the 18 isolates obtained in this study. These reference strains were selected to provide phylogenetic context for the mulberry-derived isolates and represented diverse isolation sources and previously reported ecological backgrounds. Strain names, accession numbers, isolation hosts, and geographic origins are provided in Table S1.

## **Supplementary Methods S3. Functional and antagonistic assays**

### **S3.1 Plant growth-promoting-associated functional assays**

Growth under nitrogen-free conditions was assessed on Ashby nitrogen-free agar.

Phosphate- and potassium-solubilizing activities were evaluated on Pikovskaya and Aleksandrov agar, respectively. For each assay, the solubilization index was calculated as the ratio of the clear-zone diameter to the colony diameter.

Siderophore production was assessed on chrome azurol S (CAS) agar, and formation of an orange halo around the colony was recorded as a positive reaction.

ACC utilization was evaluated using Dworkin and Foster minimal salts medium supplemented with 1-aminocyclopropane-1-carboxylate (ACC) as the sole nitrogen source. Bacterial growth was evaluated by optical-density measurement.

Indole-3-acetic acid production was estimated colorimetrically after cultivation in LB medium supplemented with L-tryptophan, followed by reaction with Salkowski reagent.

Cellulase activity was assessed on sodium carboxymethyl cellulose agar followed by Congo red staining.

### **S3.2 Additional extracellular-enzyme and hemolysis assays**

Protease activity was evaluated on skim-milk agar.

β-1,3-Glucanase activity was examined on laminarin-containing agar followed by aniline blue staining.

Hemolytic activity was assessed on Columbia blood agar.

Unless otherwise specified, functional assays were incubated at 28 ± 2°C for 24–72 h according to the requirements of the individual assay.

### **S3.3 Antagonistic activity against Ralstonia pseudosolanacearum XZqk**

Antagonistic activity against *R. pseudosolanacearum* XZqk was screened for all 18 *P. ananatis* isolates using a plate-based inhibition assay on CPG agar. Inhibition-zone diameters were recorded and used as quantitative measures of antagonistic activity.

### **S3.4 Trait classification and data integration**

For the categorical annotation tracks shown in Figure 2(a), PGPR-associated traits were classified as absent, intermediate, or strong according to assay-specific predefined thresholds.

Quantitative measurements obtained from the functional and antagonistic assays were retained for subsequent integrated phenotypic analysis together with the root-growth and branch-lesion measurements described in Sections 2.4.1 and 2.4.2.

## **Supplementary Methods S4. Root-growth, branch-explant, and integrated phenotypic analyses**

### **S4.1 Root-growth assay**

Surface-sterilized seeds of the mulberry cultivar ‘Gui Sang 12’ were immersed in 5 mL of bacterial suspension adjusted to an OD₆₀₀ of 1.0. Control seeds were immersed in the same volume of sterile medium. Treated seeds were transferred to sterile Petri dishes lined with sterile filter paper and maintained under the seedling-culture conditions described in Section 2.1.2. Primary root length was measured after 7 d. Each assay was conducted in triplicate.

### **S4.2 Branch-explant assay**

Rooted ‘Lun Jiao 40’ branch explants prepared as described in Section 2.1.2 were inoculated by stem puncture using bacterial suspensions adjusted to an OD₆₀₀ of 0.1. Control explants were punctured in the same manner and treated with sterile medium.

Leaf discoloration, wilting, vascular darkening, and lesion development were examined at 7 d post-inoculation, and lesion areas were recorded for subsequent phenotypic analysis. Each assay was conducted in triplicate.

Bacteria were re-isolated from symptomatic tissues and identified using the procedures described in Section 2.1.1 to assess their consistency with the corresponding inoculated isolates.

### **S4.3 Integrated phenotypic clustering and visualization**

An isolate-by-trait matrix was constructed by integrating quantitative PGPR-associated and antagonistic measurements with root-length and branch-lesion measurements. Each trait was standardized across the 18 isolates using Z-score transformation to account for differences in measurement scales.

Hierarchical clustering was performed using the CNSKnowall web platform with Euclidean distance and complete-linkage agglomeration. The resulting clustering pattern was used to identify the predominant phenotype-based functional groups within the isolate collection.

The Z-score-standardized isolate-by-trait matrix was visualized using the bubble-heatmap module implemented in CNSKnowall to display the relative magnitude and direction of phenotypic variation among isolates. This visualization was descriptive and was not used as an independent clustering procedure.

## **Supplementary Methods S5. Representative-strain antagonism validation and whole-plant inoculation assay**

### **S5.1 Agar-well diffusion assay**

Pa-6 and YSFJ were cultured in CPG medium for 72 h, and the resulting culture supernatants were evaluated for antagonistic activity against *Ralstonia pseudosolanacearum* XZqk using an agar-well diffusion assay. Aliquots of 20 μL of the corresponding culture supernatants were added to wells in TTC agar plates inoculated with XZqk. Sterile CPG medium served as the negative control. Inhibition-zone diameters were measured after incubation.

### **S5.2 Whole-plant inoculation assay**

Six treatments were established: CK, Pa-6, Pa-6 + XZqk, YSFJ, YSFJ + XZqk, and XZqk. Each treatment comprised 15 independent plants.

Bacterial suspensions were adjusted to approximately 10⁸ CFU mL⁻¹ and applied by root drenching after standardized root wounding. CK plants received sterile medium. Plants were maintained under the cultivation conditions described in Section 2.1.2.

Disease severity was assessed at 1, 3, 6, 9, 12, and 15 d post-inoculation using an ordinal disease-rating system adapted from Yuan et al. (2024). Representative plants were photographed at 15 d post-inoculation.

### **S5.3 Statistical analysis and visualization of disease severity**

Disease-severity trajectories were visualized using CNSKnowall. Individual disease scores, treatment means, and standard errors (SE) were displayed.

Because disease severity was recorded on a 0–5 ordinal scale, pairwise comparisons among treatments were performed independently at each observation time using two-sided Wilcoxon rank-sum tests. *P* values from pairwise comparisons within each observation time were adjusted using the Holm method, and differences were considered significant at Holm-adjusted *P* < 0.05.

## **Supplementary Methods S6. Scanning electron microscopy of bacterial cell morphology**

Purified bacterial isolates were cultured in LB medium at 28°C for 24 h. Bacterial cells were collected and fixed in a solution containing 2.5% glutaraldehyde and 4% paraformaldehyde (pH 7.0) for 3 h at 4°C.

The samples were subsequently post-fixed in 1% osmium tetroxide for 3 h, dehydrated through a graded ethanol series, critical-point dried, and sputter-coated with gold. Prepared specimens were examined using a Hitachi S-3400N scanning electron microscope at an accelerating voltage of 1–10 kV.

## **Supplementary Methods S7. Temporal symptom assessment and microscopic observation of colonization in axenic mulberry seedlings**

### **S7.1 Axenic seedling inoculation and temporal sampling**

Sixty-day-old axenic seedlings of the mulberry cultivar ‘Gui Sang 12’ (*Morus atropurpurea*) were used. Axenic status was verified as described in Section 2.1.2.

Pa-6 and YSFJ were cultured in LB medium at 28°C for 24 h. Bacterial cells were collected and suspended in sterile water, and the suspensions were adjusted to an OD₆₀₀ of 0.1. For inoculation, 10 mL of the corresponding bacterial suspension was added to the root zone of each seedling, with the roots maintained in contact with the bacterial suspension. Uninoculated control seedlings received 10 mL of sterile water and were maintained under the same aseptic conditions.

Visible symptoms, including leaf and stem yellowing, wilting, and root discoloration, were recorded at 1, 3, 5, 10, and 15 d post-inoculation (dpi). Baseline tissues were collected before inoculation, and root and stem tissues were subsequently collected at 1, 3, 5, 10, and 15 dpi.

At each sampling time, three independent seedlings were collected from each treatment. For each microscopy method, 10 microscopic fields distributed across the three biological replicates were examined for each treatment at each time point. The microscopic fields were used for qualitative assessment and selection of representative images and were not treated as independent biological replicates. Uninoculated control tissues were collected and processed in parallel.

Microscopic observations focused on bacterial presence and distribution, bacterial aggregation, starch-granule appearance, vascular organization, xylem occlusion, and cellular or tissue damage. Images presented in Figure 4 were selected as representative fields from the biological replicates examined.

### **S7.2 Scanning electron microscopy of root xylem**

Taproot xylem segments approximately 1–2 mm in length were collected and processed using the fixation, dehydration, critical-point drying, and gold-coating procedures described in Section 2.6. Prepared samples were examined using a Hitachi S-3400N scanning electron microscope.

### **S7.3 Transmission electron microscopy**

Root-tip samples were fixed in a solution containing 2.5% glutaraldehyde and 4% paraformaldehyde (pH 7.0) for 3 h at 4°C and post-fixed in 1% osmium tetroxide for 3 h.

Samples were dehydrated through a graded ethanol series, transferred through graded propylene oxide–resin mixtures, infiltrated with Spurr resin, and embedded. Ultrathin sections approximately 75 nm thick were prepared using a Leica EM UC7 ultramicrotome. Sections were stained with uranyl acetate for 15 min and lead citrate for 10 min and examined using a Hitachi HT7700 transmission electron microscope at an accelerating voltage of 80–120 kV.

### **S7.4 Paraffin-section preparation and light microscopy**

Stem segments approximately 0.5 cm in length were fixed in formalin–acetic acid–alcohol solution, softened in hydrofluoric acid for 3 d, dehydrated through a graded ethanol series, cleared in xylene, and infiltrated and embedded in paraffin.

Sections 8–10 μm thick were prepared, stained with safranin and fast green, and examined under a bright-field light microscope.

## **Supplementary Methods S8. Genome sequencing, annotation, and comparative genomic analyses**

### **Supplementary Methods S8.1. Genome sequencing, assembly, and quality assessment**

#### **S8.1.1 DNA preparation and whole-genome sequencing**

High-molecular-weight genomic DNA was extracted from pure cultures of Pa-6 and YSFJ. DNA concentration, purity, and integrity were evaluated before library construction. Short-read and long-read whole-genome sequencing were performed by Sangon Biotech Co., Ltd. (Shanghai, China).

#### **S8.1.2 Read processing and genome assembly**

Short-read sequencing data were quality filtered using Trimmomatic v0.36. Long-read sequencing data were subjected to quality assessment before genome assembly.

Quality-filtered long reads were assembled de novo using Canu v1.3. The resulting assemblies were further processed using GapFiller v1.11 to close residual gaps and PrInSeS-G v1.0.0 to correct sequence errors and short insertion–deletion events. Short-read data were subsequently used for sequence polishing with Pilon for three iterations or until no further corrections were detected.

#### **S8.1.3 Replicon circularization and assembly validation**

Complete chromosome and plasmid sequences were assessed for circularity. Circular replicons were reoriented according to predicted replication-initiation loci or, when an appropriate initiation marker was unavailable, according to GC-skew information.

Short reads were mapped back to the final assemblies to evaluate sequencing depth and genome coverage.

#### **S8.1.4 Genome-quality assessment**

Assembly quality was evaluated using genome size, replicon number, N50, sequencing depth, genome coverage, and GC content.

Genome completeness was assessed using BUSCO against conserved single-copy orthologs in the OrthoDB database, and completeness and contamination were independently assessed using CheckM based on lineage-specific marker genes.

GC-depth distributions were examined across fixed genomic bins, and circular genome maps were generated using CGView v1.0.

### **Supplementary Methods S8.2. Structural and functional genome annotation**

#### **S8.2.1 Structural annotation**

Structural annotation of the complete Pa-6 and YSFJ genome assemblies was performed using the NCBI Prokaryotic Genome Annotation Pipeline (PGAP). PGAP was used to predict protein-coding sequences, rRNA genes, tRNA genes, non-coding RNAs, and pseudogenes.

Prokka v1.10 was additionally used to generate standardized GFF annotation files for downstream comparative genomic and pan-genome analyses.

#### **S8.2.2 General functional annotation**

Predicted protein sequences were compared against the NR, COG, CDD, and Pfam databases. Similarity- and profile-based searches were performed using NCBI BLAST+ v2.2.28 and HMMER3 v3.1b1, as appropriate. Unless otherwise specified, sequence-similarity searches used an E-value threshold of 1 × 10⁻⁵.

COG and conserved-domain annotations were classified according to the corresponding database categories.

Gene Ontology annotations were obtained using eggNOG-mapper with an E-value threshold of 1 × 10⁻⁵ and were classified into biological process, cellular component, and molecular function categories.

KEGG Orthology and pathway annotations were obtained using KAAS v2.1.

#### **S8.2.3 Virulence-, resistance-, and transport-associated annotation**

Virulence-associated protein matches were identified against VFDB, whereas proteins showing sequence similarity to experimentally characterized pathogen–host interaction genes were annotated against PHI-base.

Antibiotic-resistance-associated sequence matches were identified against CARD and SARG. Biocide- and metal-resistance-associated sequence matches were identified against BacMet, and transport-related proteins were annotated against the Transporter Classification Database (TCDB).

For these sequence-similarity-based annotation resources, predicted protein sequences were searched using BLASTP with an E-value threshold of 1 × 10⁻⁵, a minimum amino-acid identity of 40%, and a minimum alignment length of 50 amino acids. These matches were interpreted as sequence-similarity-based functional annotations rather than experimental confirmation of the corresponding phenotypes.

#### **S8.2.4 Carbohydrate-active enzymes and protein-feature prediction**

Carbohydrate-active enzyme families were annotated against CAZy-derived reference data using HMMER3 v3.1b1 with an E-value threshold of 1 × 10⁻⁵.

Transmembrane helices were predicted using TMHMM v2.0, signal peptides using SignalP v4.1, lipoprotein signal peptides using LipoP v1.0, and protein subcellular localization using ProtComp v9.0.

#### **S8.2.5 Genomic islands, prophages, and biosynthetic gene clusters**

Genomic islands were predicted using IslandPath v0.2 based on dinucleotide-sequence bias and mobility-associated genes.

Prophage regions were predicted using PhiSpy v2.3, with predicted regions further compared against PHAST/PHASTER phage reference resources as implemented in the original annotation pipeline.

Biosynthetic gene clusters were predicted using antiSMASH v6.0.

### **Supplementary Methods S8.3. Comparative overlap of annotation and prediction identifiers**

Nonredundant annotation or prediction identifiers generated from the principal functional annotation resources were compared between the Pa-6 and YSFJ genomes.

Duplicate identifiers occurring within the same genome were removed before comparison. The resulting nonredundant identifier sets were used to determine identifiers shared between Pa-6 and YSFJ and identifiers associated with only one of the two genomes.

These comparisons were conducted at the annotation-ID level. Accordingly, differences in annotation identifiers were not interpreted as direct gene presence–absence differences, because multiple proteins may share the same database identifier and homologous proteins may receive different or no identifiers depending on sequence similarity and database coverage.

Detailed overlap results are presented in Figure S4 and Tables S4–S5.

### **Supplementary Methods S8.4. Average nucleotide identity and phylogenomic analyses**

#### **S8.4.1 Average nucleotide identity analysis**

Pairwise average nucleotide identity was calculated among the 41 *P. ananatis* genome assemblies using fastANI. Genome pairs for which no ANI estimate was returned were retained as missing values and were not assigned an arbitrary similarity value.

The resulting pairwise ANI matrix was visualized as a clustered heatmap. Heatmap clustering was used only for visualization and was not used for taxonomic assignment, ecological classification, or definition of functional groups.

#### **S8.4.2 Genome-derived 16S rRNA phylogeny**

The 16S rRNA phylogeny was reconstructed using 21 genomes included in the phylogenetic comparison. Genome-derived 16S rRNA gene sequences were aligned using MAFFT, and the resulting alignment was used to reconstruct the phylogenetic tree with FastTree.

#### **S8.4.3 Single-copy core-gene phylogeny**

The single-copy core-gene phylogeny was reconstructed using the same 21-genome dataset used for the corresponding phylogenetic comparison. Single-copy core-gene sequences were concatenated and aligned using MAFFT, and the resulting concatenated alignment was used for phylogenetic reconstruction with FastTree.

#### **S8.4.4 Core-genome SNP phylogeny**

Genome-wide SNP analysis was performed using the complete chromosome of the previously published mulberry-associated strain LCFJ-001 (GenBank accession CP066803) as the common reference.

Each genome was analyzed using Snippy v4.6.0. Individual Snippy outputs were subsequently combined using snippy-core to generate a core-genome SNP alignment. The resulting core-SNP alignment was used for phylogenetic reconstruction with FastTree.

#### **S8.4.5 Metadata annotation and tree visualization**

The reported categories were retained as literature-based metadata and were not treated as standardized phenotype measurements on mulberry. Geographic information was standardized at the continental level to avoid overinterpretation of heterogeneous locality information. Metadata were mapped onto the phylogenetic trees as descriptive annotations and were not used to define phylogenetic groups.

Annotated phylogenetic trees were visualized using the R package *ggtree*.

### **Supplementary Methods S8.5. Pan-genome and gene-content analyses**

#### **S8.5.1 Pan-genome construction and homologous gene-cluster classification**

Pan-genome analysis was performed using all 41 *P. ananatis* genomes with Roary. Standardized GFF files generated using Prokka were used as input, and predicted coding sequences were grouped into homologous gene clusters.

Core, shell, and cloud clusters were defined according to their distributions across the analyzed genomes. Strain-specific clusters were defined as homologous gene clusters detected in only one genome.

#### **S8.5.2 Core- and pan-genome rarefaction analysis**

Core- and pan-genome diversity was evaluated using rarefaction analyses generated from genome subsets of increasing size. For each number of included genomes, the distributions of the numbers of core and total pan-genome clusters were summarized and visualized as boxplots.

The original analysis report did not record the number of subset resampling iterations. Accordingly, no specific randomization or resampling count was assigned retrospectively.

#### **S8.5.3 COG classification of pan-genome components**

Shared and strain-specific homologous gene-cluster distributions were summarized from the Roary gene presence–absence output.

COG functional annotations were summarized separately for the core, shell, cloud, and complete pan-genome cluster sets. Category counts were calculated at the homologous gene-cluster level rather than from the total number of individual coding sequences.

#### **S8.5.4 Gene presence–absence matrix**

A separate single-copy core-gene phylogenomic tree was reconstructed using all 41 genomes in the pan-genome dataset and displayed alongside the gene presence–absence matrix. The genome order in the matrix followed the terminal order of this 41-genome tree.

For visualization, the presence–absence matrix was displayed alongside the independently reconstructed single-copy core-gene phylogenomic tree described in Section 2.8.4. Genome order followed the terminal order of the core-gene tree. No phylogenetic inference was performed directly from the gene presence–absence matrix.

#### **S8.5.5 Verification of candidate gene-content differences**

For selected strain-specific or differentially represented homologous clusters, the number and genomic positions of loci assigned to each Roary cluster were examined together with the corresponding genome annotations.

Candidate differences were assessed against the available annotations to identify cases in which related annotations or additional copies occurred in the opposite genome. Annotation-level comparisons were used to guide interpretation, but absence from an annotation or a Roary cluster was not considered sufficient to exclude all homologous sequences from the other genome

### **Supplementary Methods S8.6. Pairwise whole-genome collinearity analysis**

Pairwise whole-genome collinearity between Pa-6 and YSFJ was analyzed using MUMmer4. Pa-6 was used as the reference assembly and YSFJ as the query assembly.

Whole-genome nucleotide alignments were generated using *nucmer*. Alignment coordinates, orientations, lengths, and nucleotide identities were extracted using *show-coords*.

To summarize nucleotide similarity among the resulting collinear blocks, alignments were classified into three identity intervals: >95%, 80–95%, and <80%. For each interval, the percentage of alignment blocks was calculated by dividing the number of blocks within that interval by the total number of MUMmer alignment blocks and multiplying by 100.

These percentages therefore represent the relative frequency of alignment blocks within each nucleotide-identity interval and do not represent the proportion of the total genomic sequence length covered by that interval.

Collinear relationships between Pa-6 and YSFJ were visualized using the R package *circlize*.

## **Supplementary Methods S9. Antibiotic inhibition-zone assay and resistance-associated genomic annotation**

### **S9.1 Antibiotic disk-diffusion assay**

Antibiotic responses of the 18 mulberry-derived *P. ananatis* isolates were evaluated using antimicrobial susceptibility disks obtained from Hangzhou Binhe Microbial Reagent Co., Ltd. (Hangzhou, China).

Thirty-six antibiotics were tested, including β-lactams (cephalexin, cefuroxime, cephalothin, cefaclor, cefamandole, cefotaxime, ceftriaxone, ceftazidime, cefepime, ampicillin, amoxicillin, carbenicillin, mezlocillin, piperacillin, cefoxitin, cefmetazole, imipenem, meropenem, and aztreonam), aminoglycosides (streptomycin, kanamycin, amikacin, gentamicin, and tobramycin), tetracyclines (minocycline, tetracycline, and doxycycline), quinolones (norfloxacin, ofloxacin, and ciprofloxacin), chloramphenicol, erythromycin, spectinomycin, polymyxin, nitrofurantoin, and trimethoprim–sulfamethoxazole.

Bacterial suspensions were adjusted to an OD₆₀₀ of 0.1 and spread onto Mueller–Hinton agar. Antibiotic disks with a diameter of 5 mm were applied, and plates were incubated at 28°C for 24 h. Each isolate–antibiotic combination was tested in triplicate.

For each replicate, the 5-mm disk diameter was subtracted from the total measured inhibition-zone diameter. The three corrected values were then averaged to obtain the mean corrected inhibition-zone diameter for each isolate–antibiotic combination.

Corrected inhibition-zone diameters were operationally classified as low (≤10 mm), intermediate (>10–20 mm), or high (>20 mm) inhibition. These categories were used only for comparative analysis within the present study and were not interpreted according to clinical antimicrobial-susceptibility breakpoints.

### **S9.2 Resistance-associated genomic annotation**

Resistance-associated proteins in Pa-6 and YSFJ were annotated against CARD v3.2.5 and SARG using BLASTP.

Sequence matches were retained using an E-value threshold of ≤1 × 10⁻⁵, amino-acid identity ≥40%, and alignment length ≥50 amino acids.

Duplicate database identifiers within each genome were removed before comparison. The resulting nonredundant identifier sets were used to identify annotations shared between Pa-6 and YSFJ and annotations associated with only one of the two genomes.

CARD annotations were further summarized according to the resistance-mechanism categories recorded in the CARD/ARO metadata. As with the other sequence-similarity-based genome annotations, these matches were interpreted as resistance-associated annotations rather than experimental confirmation of the corresponding resistance mechanism.

## **Supplementary Methods S10. Untargeted metabolomics and KEGG pathway-level co-mapping**

### **Supplementary Methods S10.1. Sample preparation, LC–MS acquisition, and metabolomic data preprocessing**

#### **S10.1.1 Sample collection and preparation**

Sterile 60-day-old ‘Gui Sang 12’ mulberry seedlings were assigned to CK, Pa-6, and YSFJ treatments. Each treatment comprised three independent biological replicates, and each biological replicate was analyzed using three technical LC–MS injections.

At 15 d post-inoculation, the surrounding culture medium was collected, centrifuged at 5,000 rpm for 10 min, and filtered through a 0.20-μm PTFE membrane before untargeted metabolomic analysis.

#### **S10.1.2 LC–MS chromatographic conditions**

Metabolites were analyzed using an Agilent 6545B Q-TOF LC/MS system equipped with a Dual Agilent Jet Stream electrospray-ionization source and an SB-Aq C18 column (2.1 × 100 mm, 1.8 μm).

The injection volume was 10 μL, the flow rate was 0.3 mL min⁻¹, and the column temperature was maintained at 30°C. Mobile phase A consisted of 0.1% formic acid in water, and mobile phase B consisted of 0.1% formic acid in acetonitrile.

The gradient program was 5% B at 0–2 min, 5–95% B at 2–10 min, 95% B at 10–12 min, and 5% B at 12–15 min.

#### **S10.1.3 Mass-spectrometric conditions**

Mass spectra were acquired in both positive- and negative-ionization modes over an *m/z* range of 50–1,700 at 4 spectra s⁻¹.

The drying-gas temperature and flow rate were 300°C and 8 L min⁻¹, respectively, and the sheath-gas temperature and flow rate were 350°C and 11 L min⁻¹. Capillary voltages were 3.5 kV in positive-ion mode and 3.0 kV in negative-ion mode.

The nozzle voltage, fragmentor voltage, skimmer voltage, and octopole RF voltage were 1,000, 135, 65, and 750 V, respectively. Auto-MS/MS acquisition was performed using collision energies ranging from 10 to 40 eV.

Reference ions at *m/z* 121.0509 and 922.0098 were used in positive-ion mode, whereas *m/z* 112.9856 and 1033.9881 were used in negative-ion mode.

#### **S10.1.4 Feature extraction and data preprocessing**

Raw data were processed using Agilent MassHunter Qualitative and Quantitative Analysis v10.0 with a retention-time tolerance of ±0.2 min, a mass tolerance of ±10 ppm, and a minimum peak height of 5,000 counts.

The three technical injections corresponding to each biological replicate were averaged before downstream statistical analysis. Technical injections were therefore not treated as independent biological replicates.

The resulting feature-abundance matrix was processed in MetaboAnalyst 5.0 using sum normalization, log₂ transformation, and autoscaling.

Metabolite features consistently detected in all three CK biological replicates were removed before comparison of the Pa-6- and YSFJ-inoculated treatments.

### **Supplementary Methods S10.2. Statistical analysis, metabolite annotation, and pathway-level co-mapping**

#### **S10.2.1 Feature-level statistical analysis**

Univariate comparisons between the Pa-6- and YSFJ-inoculated treatments were performed using two-sided independent-samples *t*-tests or Wilcoxon rank-sum tests according to the original feature-level statistical analysis.

Features designated with “W” in the original analysis output were analyzed using the Wilcoxon rank-sum test, whereas the remaining features were analyzed using two-sided independent-samples *t*-tests. The resulting *P* values were adjusted across all tested features using the Benjamini–Hochberg false discovery rate procedure.

Features were assigned to Pa-6- or YSFJ-associated sets according to the direction of the mean normalized abundance difference, independently of statistical significance. Thus, assignment to either directional set did not imply that the corresponding feature met a nominal or FDR-adjusted significance threshold.

#### **S10.2.2 Putative metabolite annotation and KEGG compound mapping**

Metabolites were putatively annotated using accurate-mass matching and, where available, MS/MS spectral information against METLIN with a mass-error tolerance of <5 ppm. Putative annotations were additionally cross-referenced against PlantCyc.

Because authentic chemical standards were not available for all detected features, metabolite annotations were treated as putative identifications rather than confirmed compound identities.

Annotated metabolites were subsequently mapped to KEGG compound identifiers. When a single metabolite feature matched more than one valid KEGG compound identifier, each valid feature–compound mapping was retained as a separate entry for downstream pathway analysis.

#### **S10.2.3 Metabolite-based KEGG pathway analysis**

KEGG pathway analyses were conducted separately for the Pa-6- and YSFJ-associated compound sets using MetaboAnalyst 5.0.

Because a *P. ananatis*-specific pathway library was not available in MetaboAnalyst, the *Escherichia coli* K-12 MG1655 pathway library was used as the available bacterial reference framework. This library was used only to provide a pathway-mapping framework and was not used to infer whether the detected metabolites originated from the bacterium or the plant.

Pathway over-representation analysis was performed using the hypergeometric test, and pathway topology was evaluated using relative-betweenness centrality.

In metabolite-based pathway association networks, two pathways were connected when they shared one or more mapped KEGG compound entries.

#### **S10.2.4 Genomic–metabolomic pathway-level co-mapping**

Strain-specific homologous gene clusters identified in Section 2.8.5 were assigned KEGG Orthology (KO) identifiers.

For each strain, pathways represented by treatment-associated KEGG compound entries were compared with pathways represented by KOs derived from strain-specific homologous clusters. Pathways represented in both datasets were retained for pathway-level co-mapping.

These shared pathway assignments were interpreted as pathway co-membership. They were not considered evidence of direct physical interaction, regulatory relationships, or causal links between individual genes and metabolites.

#### **S10.2.5 Normalized pathway mapping score**

To account for differences in the total numbers of pathway-mapped entries between the genomic and metabolomic datasets, a normalized pathway mapping score was calculated separately for each pathway and dataset.

For each pathway, the number of entries assigned to that pathway was divided by the total number of pathway-mapped entries in the corresponding dataset and multiplied by 1,000.

For the metabolomic dataset, the denominator represented the total number of valid KEGG compound entries retained after one-to-many feature–compound mappings. For the genomic dataset, the denominator represented the total number of strain-specific KOs assigned to KEGG pathways.

#### **S10.2.6 Pathway-network visualization**

Pathway-level networks were visualized using Cytoscape v3.9.1.

In the integrated co-mapping networks, nodes represented KEGG pathways, compound entries, or KO entries according to the corresponding network type. Edges represented KEGG pathway membership of compounds or KOs, and edge width was proportional to the corresponding normalized pathway mapping score.

## **Supplementary Methods S11. Statistical analysis, figure preparation, and computational reproducibility**

### **S11.1 General statistical procedures**

Statistical procedures specific to individual datasets are described in the corresponding methodological subsections.

For analyses not otherwise specified, comparisons between two independent groups were performed using two-tailed independent-samples *t*-tests or non-parametric alternatives, as applicable. Comparisons involving more than two independent groups were performed using one-way analysis of variance followed by Tukey’s post hoc test where the assumptions of the analysis were satisfied.

Multiple-testing correction was applied according to the corresponding analysis. Holm adjustment was used for pairwise disease-severity comparisons within each observation time, whereas the Benjamini–Hochberg procedure was used for metabolomic feature-level comparisons.

Differences were considered statistically significant at *P* < 0.05 or FDR < 0.05, according to the corresponding analysis.

Microsoft Excel 2016 was used for data organization. Statistical analyses conducted through CNSKnowall were performed using the procedures described in the corresponding methodological subsections.

### **S11.2 Figure preparation and visualization**

Metabolomic data processing and pathway analyses were performed using MetaboAnalyst 5.0.

Venn diagrams were generated using jvenn.

The MLSA phylogenetic tree described in Section 2.2 was reconstructed in MEGA 12 using the maximum-likelihood method under the GTR+G substitution model with 1,000 bootstrap replicates and annotated using iTOL v6.

Genome-derived 16S rRNA gene, single-copy core-gene, and core-SNP phylogenetic trees described in Section 2.8.4 were reconstructed using FastTree.

Pathway-level networks were visualized using Cytoscape v3.9.1.

The graphical abstract was prepared using Figdraw, and final multi-panel figures were assembled using Adobe Illustrator 2020.

### **S11.3 Computational reproducibility**

Computational analyses were performed using the software and procedures described in the corresponding methodological sections. Custom scripts from the original analyses were not retained. Available input tables, intermediate files, analysis outputs, software versions, recorded parameters, and a computational-analysis record are provided in the public repository specified in the Data Availability section. Parameters or command-line options not preserved in the original analysis records were not reconstructed retrospectively.

# **Supplementary Results**

# **Supplementary Results SR1. Detailed molecular and phenotypic characteristics of mulberry-associated Pantoea ananatis isolates**

The 18 *P. ananatis* isolates exhibited a consistent rod-shaped morphology under scanning electron microscopy, with cell lengths of approximately 500–1,800 nm and widths of 300–600 nm (Figure S1). Combined sequence comparisons of the 16S and 23S rRNA genes, *gyrB*, *leuS*, *pleD*, and *rpoB* supported their identification as *P. ananatis*.

Multilocus sequence analysis resolved the study isolates into three principal clades. Clade I contained 11 study isolates, Clade II contained five, and Clade III contained JSFJ, whereas YZFJ occupied an intermediate position between Clades I and III. All nine publicly available reference strains included for phylogenetic context clustered within Clade III. The annotation tracks associated with the MLSA tree summarized isolate source, pathogenicity, root-length response, antagonistic activity against *Ralstonia pseudosolanacearum* XZqk, and plant growth-promoting traits.

All 18 isolates produced indole-3-acetic acid and siderophores and showed antagonistic activity against XZqk. β-1,3-Glucanase and hemolytic activities were not detected in any isolate. Growth on nitrogen-free medium, phosphate and potassium solubilization, growth on ACC-containing medium, protease activity, cellulase activity, root-length responses, and branch-lesion measurements varied among isolates.

Integrated phenotypic clustering separated the isolates into two major groups. Phenotypic Group 1 comprised 12 isolates and was generally characterized by stronger plant growth-promoting and antagonistic traits together with lower disease-associated measurements. Phenotypic Group 2 comprised six isolates and generally showed higher disease-associated measurements.

Phenotypic Group 1 comprised the 11 isolates assigned to MLSA Clade I together with JSFJ from Clade III. Phenotypic Group 2 comprised the five isolates assigned to MLSA Clade II together with YZFJ, which occupied an intermediate position between Clades I and III. Thus, most isolates assigned to the same phenotype-based group occupied similar positions in the MLSA topology, although JSFJ and YZFJ represented exceptions to the predominant pattern.

Pa-6, isolated from an asymptomatic mulberry plant, showed several plant growth-promoting and antagonistic traits together with relatively low disease-associated measurements. In contrast, YSFJ was isolated from a symptomatic mulberry plant showing whole-plant wilt and xylem blackening and exhibited comparatively high disease-associated measurements. These strains were therefore selected as representatives of the two contrasting phenotype-based groups for subsequent comparative analyses.

# **Supplementary Results SR2. Detailed disease progression following Pa-6, YSFJ, and XZqk inoculation**

The 72-h culture supernatants of both Pa-6 and YSFJ produced visible inhibition zones against *Ralstonia pseudosolanacearum* XZqk on agar plates, indicating that both strains inhibited XZqk growth under the tested in vitro conditions.

No disease symptoms were observed in any treatment at 1 day post-inoculation. Non-zero disease severity scores were first recorded in the YSFJ + XZqk and XZqk treatments at 3 dpi, followed by the YSFJ treatment at 6 dpi and the Pa-6 + XZqk treatment at 9 dpi. CK and Pa-6 treatments maintained disease severity scores of zero throughout the 15-d observation period.

Disease severity in the Pa-6 + XZqk treatment remained low during the experiment. From 9 dpi onward, disease severity scores in the Pa-6 + XZqk treatment were significantly lower than those in the XZqk-alone treatment at each examined time point (Holm-adjusted *P* < 0.05).

YSFJ alone caused progressive disease, with severe wilting and mortality evident by 15 dpi. Plants co-inoculated with YSFJ and XZqk also developed marked disease symptoms.

At 15 dpi, mean disease severity scores were 0.00 ± 0.00 for CK, 0.00 ± 0.00 for Pa-6, 0.20 ± 0.11 for Pa-6 + XZqk, 4.40 ± 0.21 for YSFJ, 4.00 ± 0.41 for YSFJ + XZqk, and 4.80 ± 0.11 for XZqk. YSFJ, YSFJ + XZqk, and XZqk did not differ significantly from one another at this time point but showed significantly higher disease severity than CK, Pa-6, and Pa-6 + XZqk (Holm-adjusted *P* < 0.05).

Thus, the shared in vitro antagonistic activity of Pa-6 and YSFJ against XZqk did not correspond to similar outcomes in the whole-plant assay.

# **Supplementary Results SR3. Detailed temporal symptom development and microscopic observations following Pa-6 and YSFJ inoculation**

Uninoculated control seedlings remained asymptomatic throughout the experiment. Their vascular tissues were regularly organized, the xylem lumina remained clear, and the examined root-cap cells contained abundant starch granules. No bacterial cells were observed in the examined control fields (Figure 4(a–d)).

At 1 dpi, seedlings inoculated with either Pa-6 or YSFJ showed no visible symptoms (Figure 4(d)). Bacterial cells were visible in the taproot xylem and vascular tissues of the YSFJ-inoculated treatment, whereas no bacterial cells were observed in the examined Pa-6 fields at this time point (Figure 4(b–c)). More starch granules were visible in root-cap cells from both inoculated treatments than in the corresponding control images (Figure 4(a)).

At 3 dpi, YSFJ-inoculated seedlings developed yellowing of the leaves and stems (Figure 4(d)). Bacterial cells were observed in root-cap tissues and taproot xylem, and fewer starch granules were visible than in the corresponding control treatment (Figure 4(a–b)). Pa-6-inoculated seedlings remained asymptomatic, although scattered bacterial cells were observed in the examined tissues (Figure 4(a–b, d)).

At 5 dpi, visible symptoms in the YSFJ-inoculated seedlings showed little additional progression relative to 3 dpi (Figure 4(d)), but bacterial cells and aggregates were more evident in root-cap tissues and xylem lumina (Figure 4(a–b)). Pa-6-inoculated seedlings remained asymptomatic, with scattered bacterial cells in the examined fields. Starch granules were not evident in the examined root-cap cells, and filamentous material was visible in the xylem lumina (Figure 4(a–b)).

By 10 dpi, YSFJ-inoculated seedlings showed extensive leaf yellowing and taproot blackening (Figure 4(d)). The examined tissues showed bacterial aggregation, marked vascular occlusion, disruption of vascular organization, and cellular damage (Figure 4(a–c)). Pa-6-inoculated seedlings showed slight stem yellowing, while bacterial cells remained comparatively scattered and no obvious vascular blockage was detected in the examined fields (Figure 4(a–d)).

At 15 dpi, YSFJ-inoculated seedlings displayed complete leaf yellowing and severe wilting (Figure 4(d)). Extensive bacterial-cell accumulation and aggregation, vascular occlusion, and cellular and tissue damage were evident in the examined microscopic fields (Figure 4(a–c)). Pa-6-inoculated seedlings showed mild leaf yellowing. Bacterial cells were more readily visible than at earlier observation times, but no obvious vascular blockage was detected, and starch granules were again visible in the examined root-cap cells (Figure 4(a–d)).

Overall, YSFJ inoculation was associated with progressive symptom development accompanied by bacterial aggregation, vascular occlusion, vascular disorganization, and tissue damage. Pa-6 inoculation was associated with substantially less tissue disruption, no obvious vascular blockage in the examined fields, and only mild yellowing at the later observation times.

# **Supplementary Results SR4. Detailed genomic and comparative genomic analyses**

#### **Supplementary Results SR4.1. Detailed sequencing and genome-assembly characteristics of Pa-6 and YSFJ**

Short-read sequencing generated high-quality datasets for both strains. Q30 proportions were 97.42% for Pa-6 and 97.35% for YSFJ, while clean reads accounted for 98.78% and 99.19% of the corresponding datasets, respectively. Long-read sequencing generated 117,303 reads for Pa-6 and 100,790 reads for YSFJ. Detailed short- and long-read sequencing statistics are provided in Tables S2–S3.

Hybrid assembly generated complete circular chromosome and plasmid sequences for both strains. Pa-6 comprised a 4,700,721-bp chromosome and one 362,399-bp plasmid, giving a total genome size of 5,063,120 bp. YSFJ comprised a 4,597,325-bp chromosome and two plasmids of 331,567 and 211,717 bp, giving a total genome size of 5,140,609 bp. The YSFJ genome was therefore 77,489 bp larger than the Pa-6 genome and contained one additional plasmid. All assembled chromosomes and plasmids were circular and gap-free (Table S3; Figure S2(a–e)).

Mean sequencing depths were 178.12× for Pa-6 and 168.29× for YSFJ, and genome coverage was 99.98% for both strains. Assembly N50 values were 4,700,721 bp for Pa-6 and 4,597,325 bp for YSFJ.

CheckM estimated 100% genome completeness for both assemblies, with contamination estimates of 1.402% for Pa-6 and 1.1% for YSFJ. BUSCO analysis likewise showed 100% completeness in both genomes. Pa-6 contained 98.39% single-copy and 1.61% duplicated BUSCOs, whereas YSFJ contained 99.19% single-copy and 0.81% duplicated BUSCOs. No fragmented or missing BUSCOs were detected in either genome.

BUSCO recovered all 124 searched BUSCO groups in Pa-6, including 122 single-copy and two duplicated groups. Sequencing-quality, GC-depth, assembly-quality, and circular-genome visualizations for both strains are shown in Figure S2(a–e).

#### **Supplementary Results SR4.2. Detailed gene prediction and functional annotation of Pa-6 and YSFJ**

Gene prediction identified 4,567 CDSs in Pa-6 and 4,729 CDSs in YSFJ. Pa-6 contained 22 rRNA genes, 78 tRNA genes, 93 predicted pseudogenes, and 22 ncRNAs, whereas YSFJ contained 22 rRNA genes, 84 tRNA genes, no predicted pseudogenes, and one ncRNA (Table 3).

Functional annotation coverage was high in both genomes. At least one functional annotation was assigned to 4,555 Pa-6 CDSs (99.74%) and 4,717 YSFJ CDSs (99.75%). NR alone provided sequence-similarity annotations for 99.72% of Pa-6 CDSs and 99.75% of YSFJ CDSs.

Additional annotation resources were used to characterize complementary functional dimensions, including conserved domains and orthology, Gene Ontology categories, metabolic pathways, carbohydrate-active enzymes, transport systems, virulence- and pathogen–host interaction-associated functions, antimicrobial- and stress-associated functions, and mobile or specialized genomic features.

Despite differences in the absolute numbers of some database-specific annotations, the overall functional-category distributions of Pa-6 and YSFJ were broadly similar. Complete database-specific annotation counts and category distributions are provided in Table 3, Tables S4–S5, and Figure S3.

#### **Supplementary Results SR4.3. Detailed comparison of database annotation identifiers between Pa-6 and YSFJ**

Comparison of nonredundant annotation and prediction identifiers showed extensive overlap between Pa-6 and YSFJ across the examined functional resources (Figure S4(a–g); Tables S4–S5). The shared component generally exceeded the strain-associated component, consistent with the high overall similarity of the two genomes and their broadly comparable functional-annotation profiles.

Differences were more evident in several specialized annotation resources (Figure S4(h–k)). In the TCDB comparison, Pa-6 and YSFJ contained 20 and 22 strain-associated annotation IDs, respectively. The corresponding numbers were 16 and 29 for PHI-base, 5 and 12 for VFDB Set A, and 65 and 74 for VFDB Set B. Thus, YSFJ contained somewhat larger strain-associated annotation-ID sets in the pathogen–host interaction and virulence-related databases, whereas the transport-associated comparison was more similar between the two genomes.

These values represent nonredundant database annotation identifiers rather than unique genes. Accordingly, differences in identifier counts were not interpreted as direct gene-content differences. Homologous gene-cluster and locus-level analyses described in Section 3.4.6 were instead used as the primary basis for identifying complete gene presence–absence differences between Pa-6 and YSFJ.

Resistance-associated annotation overlap was analyzed separately using CARD and is described in Section 3.4.4 and Figure S4(l).

#### **Supplementary Results SR4.4. Detailed antibiotic inhibition-zone profiles and resistance-associated annotations**

The 18 mulberry-derived *P. ananatis* isolates exhibited heterogeneous inhibition-zone profiles across the 36 antibiotics tested (Figure S5(a-b)). Based on corrected inhibition-zone diameters, responses were operationally classified as low, intermediate, or high inhibition. The distribution of these response categories varied among antimicrobial compounds and, for several antibiotics, among individual isolates. Complete corrected inhibition-zone measurements and corresponding response classifications are provided in Figure S5 and the associated supplementary dataset.

CARD-based comparison of Pa-6 and YSFJ identified 109 shared nonredundant resistance-associated annotation IDs, together with four Pa-6-associated and one YSFJ-associated annotation IDs (Figure S4(l); Tables S4–S6).

The shared annotation set included multiple resistance-associated functions. Representative matches included *emrA*, *emrB*, *acrB*, and *tolC* associated with antibiotic efflux; *vanG* and *arnA* associated with target alteration; *cmlv* and *FONA-5* associated with antibiotic inactivation; and *dfrA3* and *sul4* associated with target replacement.

Among selected strain-associated annotations, a *salA*-matched annotation was detected in Pa-6, whereas *carA*- and *Kpne_KpnH*-related annotations were detected in YSFJ (Tables S4–S6). These selected differences did not show a simple correspondence with the relative inhibition-zone patterns observed for the two strains.

Overall, the large shared CARD-annotation component indicated that most detected resistance-associated functions were common to Pa-6 and YSFJ, whereas the strain-associated component represented only a small fraction of the resistance-related annotation profiles.

#### **Supplementary Results SR4.5. Detailed ANI and phylogenomic relationships among the 41 Pantoea ananatis genomes**

Comparative genomic and phylogenomic analyses included 41 *P. ananatis* genomes: 38 publicly available reference genomes, the previously published mulberry-associated strain LCFJ-001, and the two mulberry-associated strains Pa-6 and YSFJ sequenced in the present study (Table S7).

Pairwise ANI analysis showed generally high nucleotide identity among the analyzed genomes (Figure 5(a)). Among the three mulberry-associated strains, Pa-6 and LCFJ-001 showed the highest reciprocal ANI values, ranging from 99.0916% to 99.1111%. Reciprocal ANI values ranged from 98.9870% to 99.0003% between YSFJ and LCFJ-001 and from 98.9126% to 98.9369% between Pa-6 and YSFJ.

The phylogenomic tree inferred from concatenated single-copy core-gene sequences placed Pa-6 and YSFJ in two different major clades (Figure 5(b)). LCFJ-001 was positioned on a separate branch outside the two highlighted clades. Visual comparison with the associated metadata did not reveal an obvious correspondence between the major phylogenomic groupings and the reported ecological or functional category, host or isolation source, or geographical origin of the analyzed genomes.

The 16S rRNA gene phylogeny placed Pa-6, YSFJ, and LCFJ-001 at distinct positions rather than within a single mulberry-associated cluster (Figure 5(c)). Similarly, the three strains did not form a single group in the core-genome SNP phylogeny. In the SNP-based tree, Pa-6 and LCFJ-001 occurred within the same broader clade, whereas YSFJ was positioned in a different clade (Figure 5(d)).

Overall, the relative positions of the three mulberry-associated strains differed among the ANI, single-copy core-gene, 16S rRNA, and core-genome SNP analyses. Pa-6 and LCFJ-001 showed the highest ANI values among the three focal strains and occurred within the same broader clade in the SNP phylogeny, whereas Pa-6 and YSFJ were separated into different major clades in the single-copy core-gene phylogeny.

#### **Supplementary Results SR4.6. Detailed pan-genome, gene-content, and collinearity analyses**

Pan-genome analysis of the 41 *P. ananatis* genomes identified 15,201 homologous gene clusters, comprising 3,511 core, 1,263 shell, and 10,427 cloud clusters (Figure 6(c); Table S8). Rarefaction analysis showed a progressive increase in the number of pan-genome clusters as additional genomes were included, whereas the number of core clusters decreased and tended toward stabilization (Figure 6(a)).

The petal analysis showed 3,511 core gene clusters shared across the analyzed genomes together with substantial variation in the numbers of strain-specific clusters (Figure 6(b)). YSFJ contained 150 strain-specific homologous clusters, whereas Pa-6 contained 209.

Examination of the strain-specific homologous clusters together with the corresponding genome annotations identified a limited set of clear gene-level presence–absence differences between Pa-6 and YSFJ. A GH19 glycoside-hydrolase locus was detected in Pa-6 but not in YSFJ, whereas *wbpZ* and *feoB* were detected in YSFJ but not in Pa-6 (Tables S4, S5, and S8). Several other genes represented by strain-specific homologous clusters were also represented by related sequences or additional copies in the opposite genome and were therefore treated as sequence- or copy-level differences rather than complete gene-level presence–absence differences.

COG classification showed different functional distributions among the major pan-genome components. Within the cloud gene-cluster set, function unknown and transcription were the largest categories, comprising 416 and 330 annotated clusters, respectively (Figure 6(d)). Within the core gene-cluster set, amino acid transport and metabolism, carbohydrate transport and metabolism, and transcription were the largest categories, comprising 319, 283, and 232 clusters, respectively (Figure 6(e)).

Across the complete pan-genome, general function prediction only and function unknown were the most highly represented COG categories, comprising 911 and 750 annotated clusters, respectively (Figure 6(f)). Within the shell gene-cluster set, the corresponding two categories were also the largest, comprising 75 and 71 clusters, respectively (Figure 6(g)).

The gene presence–absence matrix showed extensive variation in homologous-cluster distribution across the 41 genomes (Figure 6(h)). In the accompanying independently reconstructed single-copy core-gene phylogenomic tree, Pa-6 was positioned adjacent to *P. ananatis* NN08200 and YJ76, whereas YSFJ was positioned near *P. ananatis* BRT175. The phylogenomic tree was used to order genomes in the presence–absence matrix and was not inferred from the gene-content data.

Pairwise whole-genome collinearity analysis between Pa-6 and YSFJ identified 2,440 alignment blocks (Figure 6(i)). Of these, 51.51% showed nucleotide identities greater than 95%, 42.71% showed identities between 80% and 95%, and 5.78% showed identities below 80%. These percentages represent the proportions of identified alignment blocks within the corresponding nucleotide-identity intervals and not the proportions of total genomic sequence length.

# **Supplementary Results SR5. Detailed host-contact metabolomic analyses**

#### **Supplementary Results SR5.1. Detailed feature detection, normalization, and global metabolic variation among CK, Pa-6, and YSFJ treatments**

Across the three biological replicates, 103–122, 152–181, and 193–239 metabolic features were detected in the CK, Pa-6, and YSFJ treatments, respectively, in positive-ionization mode. In negative-ionization mode, the corresponding ranges were 83–119, 189–246, and 228–284 features. Thus, both inoculated treatments contained more detectable metabolic features than CK, with the highest feature counts observed in YSFJ in both ionization modes. Differences in the overall chromatographic profiles among the treatments were also evident in the total-ion chromatograms (Figure S6(a–f); Tables S9–S10).

The overall intensity distributions of detected metabolic features were examined before and after normalization. In both positive- and negative-ionization modes, normalization reduced differences in sample-wise signal distributions and brought the overall intensity ranges into closer agreement, as shown by the density distributions and boxplots (Figure S7(a–b)).

Principal-component analysis showed treatment-associated variation in both ionization modes. In positive-ionization mode, PC1 and PC2 explained 20.7% and 17.8% of the total variance, respectively, corresponding to a combined 38.5%. CK, Pa-6, and YSFJ occupied distinguishable regions of the score plot, although the YSFJ replicates showed greater within-treatment dispersion than the CK and Pa-6 replicates (Figure S7(c)).

In negative-ionization mode, PC1 and PC2 explained 30.4% and 17.2% of the total variance, respectively, corresponding to a combined 47.6%. CK samples were separated from the inoculated treatments, whereas Pa-6 and YSFJ showed partial overlap (Figure S7(d)).

Exploratory partial least-squares discriminant analysis also showed separation among CK, Pa-6, and YSFJ in both positive- and negative-ionization modes (Figure S7(e–f)). Because each treatment comprised only three independent biological replicates, these supervised analyses were interpreted as exploratory visualizations rather than as independent confirmation of treatment separation.

Sample-correlation analyses showed high similarity among CK replicates, whereas the Pa-6 and YSFJ treatments showed treatment-associated correlation patterns together with greater within-treatment variation (Figure S7(g–h)).

Hierarchical-clustering heatmaps further showed treatment-associated feature-abundance patterns in both ionization modes, although overlap among the three treatments remained evident for subsets of metabolic features (Figure S7(i–j)).

Overall, the global metabolomic analyses indicated that inoculation with Pa-6 or YSFJ was associated with changes in the extracellular metabolic profiles of the mulberry seedling culture system, while also showing within-treatment variability and partial overlap between the two inoculated treatments.

#### **Supplementary Results SR5.2. Detailed inoculation-associated and treatment-specific metabolic features**

Eighteen nonredundant metabolic features detected in the inoculated treatments but excluded from the CK-associated feature set were putatively annotated. Representative annotations included choline, pantothenic acid, N-(3-oxo-hexanoyl)-homoserine lactone, 1,5-anhydro-D-fructose, 3-isopropylmalate, leucinic acid, kaempferol 3-(6G-malonylneohesperidoside), 2,6,7,4′-tetrahydroxyisoflavanone, and chalcomoracin. The complete set of putative annotations and corresponding molecular formulas is provided in Table 4.

Five features were detected only in the Pa-6-inoculated treatment. These were putatively annotated as hydroxyprolyl-threonine, bidenoside B, bis(2-hydroxyethyl)dithiocarbamic acid, 3-hydroxy-4-butanolide, and (S)-3-octanol glucoside.

Four features were detected only in the YSFJ-inoculated treatment. These were putatively annotated as trans-S-(1-propenyl)-L-cysteine, 5-(3′,5′-dihydroxyphenyl)-γ-valerolactone 3-O-glucuronide, methazolamide, and 3,4-dehydrothiomorpholine-3-carboxylate.

Because the analyzed samples consisted of culture medium collected from mulberry seedlings after inoculation, these features were classified according to treatment-associated detection patterns. Their detection does not establish whether an individual compound originated directly from bacterial metabolism, plant metabolism, or the plant–bacterium interaction.

#### **Supplementary Results SR5.3. Detailed relative-abundance differences between the Pa-6- and YSFJ-inoculated treatments**

Comparison of the Pa-6- and YSFJ-inoculated treatments identified 335 metabolic features showing different directions of mean normalized abundance across the positive- and negative-ionization datasets. Among these, 165 features showed higher mean normalized abundance in the Pa-6 treatment, whereas 170 showed higher mean normalized abundance in the YSFJ treatment (Figure S8(a–f); Table S11).

These two feature sets were defined according to the direction of the mean abundance difference and independently of statistical significance. Accordingly, assignment to the Pa-6- or YSFJ-associated set did not indicate that an individual feature met either the nominal or FDR-adjusted significance threshold.

Eighteen features showed nominal evidence of differences between the two treatments (*P* < 0.05). Twelve of these showed higher mean normalized abundance in Pa-6, whereas six showed higher mean normalized abundance in YSFJ.

After Benjamini–Hochberg correction across all tested features, three remained significant at FDR < 0.05. Two features showed higher mean abundance in the Pa-6 treatment: C₂₄H₃₆O₅ (FDR = 0.00335) and C₅H₁₁NO₂S₂ (FDR = 0.01563). One feature, C₅H₈N₄O₃S₂ (FDR = 0.00503), showed higher mean abundance in the YSFJ treatment.

The remaining 15 nominally different features did not meet the FDR < 0.05 threshold after multiple-testing correction. Complete feature-level information, including treatment-associated abundance direction, mean normalized intensity, statistical test, nominal *P* value, and Benjamini–Hochberg-adjusted *P* value, is provided in Table S11.

Fold-change and volcano-plot visualizations summarized the distribution of features showing higher relative abundance in Pa-6 or YSFJ across the positive- and negative-ionization datasets (Figure S8(a–f)).

#### **Supplementary Results SR5.4. Detailed KEGG pathway analysis and pathway-level co-mapping of Pa-6-associated metabolic features**

The 165 metabolic features showing higher mean normalized abundance in culture supernatants from Pa-6-inoculated seedlings than in those from YSFJ-inoculated seedlings were subjected to KEGG pathway analysis after compound annotation. The resulting compound assignments highlighted the citrate cycle, alanine, aspartate and glutamate metabolism, other carbon fixation pathways, butanoate metabolism, nicotinate and nicotinamide metabolism, and tyrosine metabolism (Figure 7(a)). Additional represented pathways included glyoxylate and dicarboxylate metabolism, pyruvate metabolism, arginine biosynthesis, arginine and proline metabolism, lysine degradation, β-alanine metabolism, sulfur metabolism, and terpenoid backbone biosynthesis.

The metabolite-based pathway association network showed extensive links among pathways involved in central carbon metabolism, amino-acid metabolism, organic-acid metabolism, and cofactor-related metabolism (Figure 7(b)). The citrate cycle was connected to pyruvate metabolism, glyoxylate and dicarboxylate metabolism, butanoate metabolism, and other carbon fixation pathways. Alanine, aspartate and glutamate metabolism was connected to butanoate metabolism, arginine biosynthesis, arginine and proline metabolism, and β-alanine metabolism. Nicotinate and nicotinamide metabolism and tyrosine metabolism were also linked to multiple pathways.

Pathway-level co-mapping identified 25 KEGG pathway categories jointly represented by Pa-6-associated KEGG compound entries and KOs derived from Pa-6-specific homologous clusters (Figure 7(c-d)). Among the broad KEGG categories, metabolic pathways (map01100) showed normalized pathway mapping scores of 124.14 for the metabolomic dataset and 137.93 for the genomic dataset. Biosynthesis of secondary metabolites (map01110) showed corresponding scores of 55.17 and 63.22, whereas microbial metabolism in diverse environments (map01120) showed scores of 41.38 and 63.22, respectively.

Among the more specific jointly represented pathways, glycolysis/gluconeogenesis (map00010) contained arbutin (C06186) together with K01610, annotated as *pckA*. Alanine, aspartate and glutamate metabolism (map00250) contained 4-aminobutanoate (C00334) together with K01744, annotated as *aspA*. Butanoate metabolism (map00650) contained 4-aminobutanoate and maleic acid (C01384), together with K00074, K00626, K01034, and K01035, corresponding to enzymes involved in short-chain acyl-CoA metabolism.

Nicotinate and nicotinamide metabolism (map00760) included 4-aminobutanoate, maleic acid, and 1-methylnicotinamide (C02918), together with K00324 and K00325, corresponding to the proton-translocating transhydrogenase subunits PntA and PntB.

UDP-glucose (C00029) was assigned to amino sugar and nucleotide sugar metabolism (map00520), biosynthesis of various nucleotide sugars (map00541), and biosynthesis of nucleotide sugars (map01250). These pathways were jointly represented with K00973 and additional orthologs involved in dTDP-sugar biosynthesis.

Other jointly represented pathways included fatty acid degradation, ubiquinone and other terpenoid-quinone biosynthesis, purine metabolism, tryptophan metabolism, thiamine metabolism, terpenoid backbone biosynthesis, phenylpropanoid biosynthesis, biosynthesis of ansamycins, degradation of aromatic compounds, biosynthesis of amino acids, nucleotide metabolism, biosynthesis of cofactors, ABC transporters, and quorum sensing.

The integrated pathway-mapping network summarized the relative representation of these 25 shared pathways in the metabolomic and genomic datasets (Figure 7(c)), whereas the core co-mapping network showed the specific KEGG compound and KO entries assigned to each jointly represented pathway (Figure 7(d)). These connections indicate shared pathway membership only and do not establish direct regulatory or causal relationships between individual genes and metabolites. Complete normalized pathway mapping scores and pathway-to-node assignments are provided in Tables S12 and S13, respectively.

#### **Supplementary Results SR5.5. Detailed KEGG pathway analysis and pathway-level co-mapping of YSFJ-associated metabolic features**

The 170 metabolic features showing higher mean normalized abundance in culture supernatants from YSFJ-inoculated seedlings than in those from Pa-6-inoculated seedlings yielded 214 KEGG compound entries after retention of valid one-to-many feature–compound mappings. Pathway analysis highlighted glycerophospholipid metabolism, pantothenate and CoA biosynthesis, one-carbon pool by folate, and glycine, serine and threonine metabolism (Figure 8(a)). Additional represented pathways included β-alanine metabolism, purine metabolism, lysine biosynthesis, glyoxylate and dicarboxylate metabolism, xylene degradation, benzoate degradation, aminobenzoate degradation, ubiquinone and other terpenoid-quinone biosynthesis, folate biosynthesis, and porphyrin metabolism.

The metabolite-based pathway association network showed links among pathways involving amino-acid, lipid, cofactor, purine, and aromatic-compound metabolism (Figure 8(b)). Glycine, serine and threonine metabolism was connected to glycerophospholipid metabolism and one-carbon pool by folate, whereas pantothenate and CoA biosynthesis was linked to β-alanine metabolism. Benzoate degradation, aminobenzoate degradation, and ubiquinone and other terpenoid-quinone biosynthesis formed an additional connected group.

Pathway-level co-mapping identified 29 KEGG pathway categories jointly represented by YSFJ-associated KEGG compound entries and KOs derived from YSFJ-specific homologous clusters (Figure 8(c-d)). Among the broad KEGG categories, metabolic pathways (map01100) showed normalized pathway mapping scores of 144.86 for the metabolomic dataset and 147.73 for the genomic dataset. Biosynthesis of secondary metabolites (map01110) showed corresponding scores of 74.77 and 79.55, whereas microbial metabolism in diverse environments (map01120) showed scores of 56.07 and 56.82, respectively.

Among the more specific jointly represented pathways, ABC transporters (map02010) showed normalized pathway mapping scores of 32.71 for the metabolomic dataset and 22.73 for the genomic dataset. This pathway included sucrose (C00089), lactose (C00243), uridine (C00299), deoxyadenosine (C00559), betaine (C00719), D-allose (C01487), and xanthosine (C01762), together with K05658, K09690, K09691, and K10037.

Carbon metabolism (map01200) included phosphoenolpyruvate (C00074), 2-phospho-D-glycerate (C00631), and the orthologs K00018, K00058, K00248, K01689, K01834, and K03781. Several jointly represented pathways were associated with carbohydrate transport and utilization. The phosphotransferase system (map02060) included phosphoenolpyruvate, sucrose, lactose, and the glucitol/sorbitol PTS orthologs K02781 and K02783. Fructose and mannose metabolism (map00051) contained D-allose together with K00008, K01711, K01840, K02781, and K02783. Galactose metabolism (map00052) included sucrose, lactose, and K01784, whereas glycolysis/gluconeogenesis (map00010) included phosphoenolpyruvate, 2-phospho-D-glycerate, K01689, and K01834.

Pathways associated with amino-acid metabolism and aromatic-compound degradation were also jointly represented. Glyoxylate and dicarboxylate metabolism (map00630) included 2-phospho-D-glycerate together with K00018, K01569, and K03781. Lysine biosynthesis (map00300) contained N-succinyl-2-L-amino-6-oxoheptanedioate (C04462) and K01586. Benzoate degradation (map00362) included 2,5-dihydroxybenzoate (C00628) and K01607, whereas degradation of aromatic compounds (map01220) included toluate (C01454), 2-oxo-5-methyl-*cis*-muconate (C07479), *cis*-3-(3-carboxyethenyl)-3,5-cyclohexadiene-1,2-diol (C12622), and K01607.

Overall, the 29 jointly represented pathway categories included functions associated with central carbon metabolism, carbohydrate transport and utilization, amino-acid metabolism, cofactor biosynthesis, membrane transport, and aromatic-compound degradation (Figure 8(c-d)). These shared pathway assignments indicate pathway co-membership only and do not establish direct regulatory or causal relationships between individual genes and metabolites. Complete normalized pathway mapping scores and pathway-to-node assignments are provided in Tables S14 and S15, respectively.
